# Moss-produced human complement factor H with modified glycans has an extended half-life and improved biological activity

**DOI:** 10.3389/fimmu.2024.1383123

**Published:** 2024-05-10

**Authors:** Todor Tschongov, Swagata Konwar, Andreas Busch, Christian Sievert, Andrea Hartmann, Marina Noris, Sara Gastoldi, Sistiana Aiello, Andreas Schaaf, Jens Panse, Peter F. Zipfel, Paulina Dabrowska-Schlepp, Karsten Häffner

**Affiliations:** ^1^ Department of Internal Medicine IV (Nephrology), Medical Center, Faculty of Medicine, University of Freiburg, Freiburg, Germany; ^2^ Eleva GmbH, Freiburg, Germany; ^3^ Department of Infection Biology, Leibniz Insitute for Natural Product Research and Infection Biology, Jena, Germany; ^4^ Centro di Ricerche Cliniche per le Malattie Rare “Aldo e Cele Dacco”, Istituto di Ricerche Farmacologiche Mario Negri IRCCS, Bergamo, Italy; ^5^ Department of Oncology, Hematology, Hemostaseology and Stem Cell Transplantation, University Hospital Rheinisch-Westfälische Technische Hochschule (RWTH) Aachen, Aachen, Germany; ^6^ Center for Integrated Oncology (CIO), Aachen, Bonn, Cologne, Düsseldorf (ABCD) Germany Pauwelsstrasse 30, Aachen, Germany; ^7^ Institute of Microbiology, Friedrich-Schiller-University, Jena, Germany

**Keywords:** recombinant factor H, C3 glomerulopathy, complement-associated disease, CPV-104, CFH, complement

## Abstract

Most drugs that target the complement system are designed to inhibit the complement pathway at either the proximal or terminal levels. The use of a natural complement regulator such as factor H (FH) could provide a superior treatment option by restoring the balance of an overactive complement system while preserving its normal physiological functions. Until now, the systemic treatment of complement-associated disorders with FH has been deemed unfeasible, primarily due to high production costs, risks related to FH purified from donors’ blood, and the challenging expression of recombinant FH in different host systems. We recently demonstrated that a moss-based expression system can produce high yields of properly folded, fully functional, recombinant FH. However, the half-life of the initial variant (CPV-101) was relatively short. Here we show that the same polypeptide with modified glycosylation (CPV-104) achieves a pharmacokinetic profile comparable to that of native FH derived from human serum. The treatment of FH-deficient mice with CPV-104 significantly improved important efficacy parameters such as the normalization of serum C3 levels and the rapid degradation of C3 deposits in the kidney compared to treatment with CPV-101. Furthermore, CPV-104 showed comparable functionality to serum-derived FH *in vitro*, as well as similar performance in *ex vivo* assays involving samples from patients with atypical hemolytic uremic syndrome, C3 glomerulopathy and paroxysomal nocturnal hematuria. CPV-104 – the human FH analog expressed in moss – will therefore allow the treatment of complement-associated human diseases by rebalancing instead of inhibiting the complement cascade.

## Introduction

The complement system is a major humoral component of the innate immune response, featuring a complex network of plasma and membrane-associated proteins. Its main functions are to recognize, destroy and remove invading pathogens, apoptotic cells, immune complexes, and damaged host cells (1, 2). Beyond these classical, systemic roles, the complement system also has important non-canonical functions such as synaptic pruning (3), T-cell differentiation (4), B-cell antibody production (5), and the control of basic cellular processes via intracellular complement, otherwise known as the complosome (6). The depletion or dysfunction of complement components can lead to uncontrolled complement activation, manifesting in several human diseases such as atypical hemolytic uremic syndrome (aHUS) (7), C3 glomerulopathy (C3G) (8, 9), IgA nephropathy (10), paroxysomal nocturnal hemoglobinuria (PNH) (11), autoimmune conditions (12) and many more (13).

Given the broad range of complement-associated disorders, the complement system is increasingly recognized as an important therapeutic target. Until recently, the only approved treatments were the humanized monoclonal antibodies eculizumab (Soliris) and ravulizumab (Ultomiris) targeting C5. Eculizumab was approved for the treatment of PNH in 2007 (14) and aHUS in 2011 (15), whereas ravulizumab (a longer-lasting derivative of eculizumab) was approved in 2018 (16). However, the number of complement-targeting drugs and drug candidates in the development pipeline has increased significantly over the last few years (17), with prominent examples including the C3 inhibitor pegcetacoplan (Aspaveli/Empaveli/Syfovre) for the treatment of PNH (18) and geographic atrophy (19, 20), the C1s inhibitor sutimlimab (Enjaymo) for cold agglutinin disease (21), the C1-esterase inhibitor (Cinryze) for hereditary angioedema (HAE) (22), the C5aR1 inhibitor avacopan (Tavneos) for anti-neutrophil cytoplasmic antibody-associated vasculitis (23), and the most recently approved factor B inhibitor iptacopan (Fabhalta) for PNH (24).

Importantly, most present-day complement therapies elicit a complete blockade of the complement system or the activation effector pathway, thereby preventing opsonization by C5 and blocking the membrane attack complex (MAC). Accordingly, patients treated with eculizumab have a significantly higher risk of life-threatening infections caused by *Neisseria meningitidis* and other pathogenic bacteria, including *Haemophilus influenzae* and *Streptococcus pneumoniae* (25, 26). Another concern is the risk of poor patient compliance, which can reduce efficacy particularly when using proximal complement inhibitors due to the more profound manifestation of breakthrough hemolysis (BTH). This is because the escape of a single C5 molecule from treatment with a terminal C5 inhibitor can trigger the formation of only one MAC, whereas the escape of a single C3 molecule can give rise to a C5 convertase, which in turn can generate multiple downstream MACs, thus promoting breakthrough events (27).

**Figure 1:**
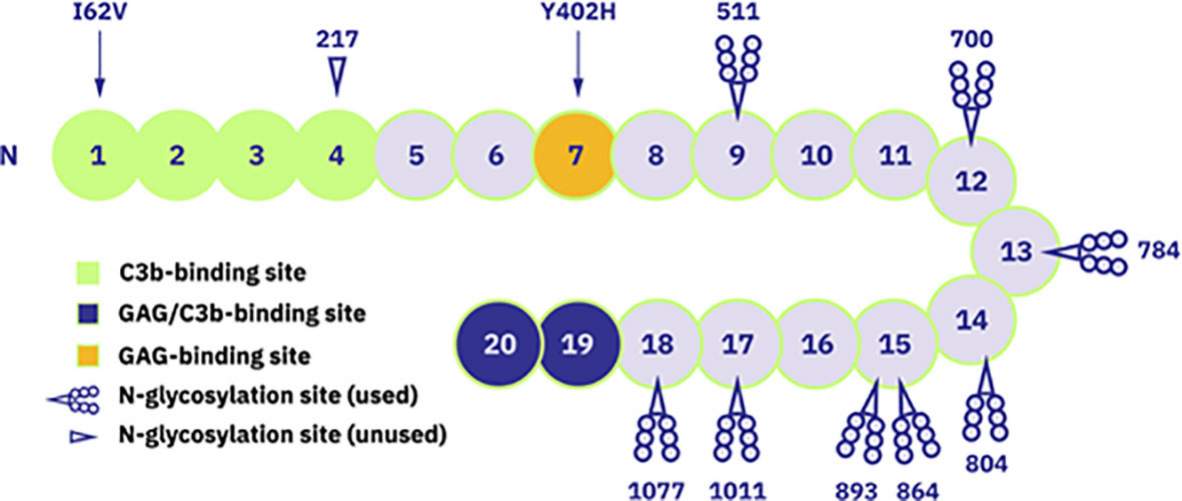
Schematic representation of FH structure with 20 short consensus repeat (SCR) domains. Domains with known functions are marked: SCR1-4 as C3b-binding site, SCR19-20 as glycosyaminoglycan (GAG)- and C3b-binding site and SCR7 as GAG-binding site. Additionally, the occupied and unoccupied glycosylation sites of FH protein are indicated as well as locations of mentioned polymorphism variants (Ile/Val62 and His/Tyr402). Figure adapted from (30). Copyright (2011), with permission from Elsevier.

Naturally occurring complement regulators such as factor H (FH) can provide more elegant and even superior treatment options by restoring the balance of a deregulated pathogenic complement system while preserving its normal physiological functions. FH is an abundant human plasma protein composed of 20 short consensus repeat (SCR) domains also known as complement control protein modules (CCPs) (28, 29) (**Figure 1**). FH acts as a cofactor for factor I, which cleaves surface-bound C3b molecules into inactive iC3b (31). FH also increases decay-accelerating activity by displacing factor Bb from C3b molecules, destabilizing the pre-formed C3 convertase (28, 32). SCR19-20 domains bind to the host cell surface, with high specificity for sialic acid residues and heparan sulfates, thus distinguishing between self and non-self, balancing complement action, and preventing unwanted complement activation on host cells (28, 33).

Given the key regulatory role of FH, mutations in the human *CFH* gene and/or the presence of anti-FH autoantibodies have been described in several pathological conditions. For example, aHUS and C3G are associated with mutations mainly clustering in the SCR19-20 and SCR1-4 domains of *CFH*, respectively (34, 35), as well as the presence of anti-FH autoantibodies (36, 37). Many *CFH* polymorphisms have also been described as causative or risk factors for various diseases (38, 39). The best-known example is the common polymorphism 402H, a major genetic risk factor for age-related macular degeneration (AMD) (40–43).

The serum concentration of FH is ~500 mg/L in healthy individuals, and the systemic treatment of complement-related disorders with FH would therefore require large amounts of protein. Due to the high costs and risks related to FH obtained from donors’ blood, systemic treatment with purified FH has been deemed unfeasible. The production of functional recombinant FH is also challenging (44, 45). The highest yields thus far have been achieved in yeast (30), but the recombinant protein lacked *N*-linked glycans and this substantial difference compared to native FH raised concerns about immunogenicity and a limited serum half-life (46). Human FH has also been expressed in human embryonic kidney (HEK) cells for the local treatment of AMD (47, 48). This recombinant FH (GEM-103) was evaluated in phase I (NCT04246866) and II (NCT04643886) clinical trials in AMD patients (49) but failed to achieve its clinical end points and the developmental program was terminated (50).

Here we present an alternative, successful approach to generate human FH for therapeutic purposes. We have previously shown that the moss *Physcomitrium patens* is an efficient expression system for the production of functional recombinant FH with high yields (51). The first moss-produced FH variant (CPV-101) behaved similarly to serum-derived FH (sd-FH) *in vitro* and normalized low serum C3 levels and dissolved pathologic C3 deposits in a mouse model of C3G. However, the serum half-life of this variant was significantly shorter than that of its human counterpart (51). Short circulation times limit the use of recombinant biotherapeutics by reducing systemic bioavailability (52). This can sometimes be overcome by shorter dosing intervals, higher doses, alternative administration routes or controlled release, but these approaches often result in higher costs and patient compliance issues. The serum half-life of biotherapeutics can be extended by chemical modification, such as conjugation to polyethylene glycol (PEG) to increase the protein’s hydrodynamic volume, or the addition of Fc-IgG domains or albumin. However, the limited half-life of biotherapeutics is often caused by incorrect glycosylation, particularly the lack of sialylation (53–55). Increasing the sialic acid content of glycans can therefore enhance the pharmacokinetic properties of recombinant therapeutic proteins (54, 56, 57).

Human FH contains eight occupied *N*-glycosylation sites, most of which are diantennary disialylated glycans (58) (**Figure 1**). In contrast, 45–60% of the *N*-linked glycans in CPV-101 feature terminal *N*-acetylglucosamine (GlcNAc/GlcNAc) residues. Aiming to increase serum half-life of FH, we therefore capped these terminal GlcNAc residues with sialic acid (Neu5Ac) and generated the variant CPV-104. This glycosylation optimized protein displayed all relevant complement regulatory functions *in vitro*, and at the same time demonstrated superior pharmacokinetic and pharmacodynamic properties *in vivo* in mice and primates compared to the original CPV-101. The successful production of therapeutically useful quantities of a new, improved human FH analog (CPV-104) in moss will allow the treatment of pathologic complement conditions by rebalancing the defective complement system rather than inducing a complete therapeutic blockade.

## Materials and methods

### Reference proteins and sera

Complement proteins (FH, C3b, FI, C3, FB and FD) as well as FH-depleted serum were obtained from Complement Technologies (USA). CVF was purchased from Quidel (USA). Eculizumab and pegcetacoplan were obtained from remnants of infusions. Blood from aHUS patients and healthy volunteers was collected through venipuncture and allowed to clot for 30 min before centrifugation for 10 min at 2.000 G. Serum was aliquoted into cryotubes and frozen immediately at -80°C until experiments were performed. For each repeated experiment involving normal human serum, sera from different healthy volunteers were used. Blood from PNH patients was collected into EDTA tubes and stored for 24 h before the experiments were performed. In this study, we used samples from two groups of aHUS patients: two patients (aHUS1 and aHUS2) treated at the Medical Center, University of Freiburg (study approved by the Ethics Committee of Freiburg, 368/10) and six patients (aHUS3–aHUS8) from the Mario Negri Institute in Bergamo (study approved by the Ethics Committee of Azienda Socio-Sanitaria Territoriale Papa Giovanni XXIII). For *in vitro* experiments with PNH erythrocytes, we used samples from four PNH patients treated at the University Hospital RWTH Aachen (PNH1–PNH4). Blood was taken via venipuncture into EDTA containing tubes and stored at 4°C 24 h before the experiments were performed. All patients included in this study provided informed written consent. Patient details are provided in the **Supplementary Material**.

### Production and purification of CPV-104 and CPV-101

**Figure 2:**
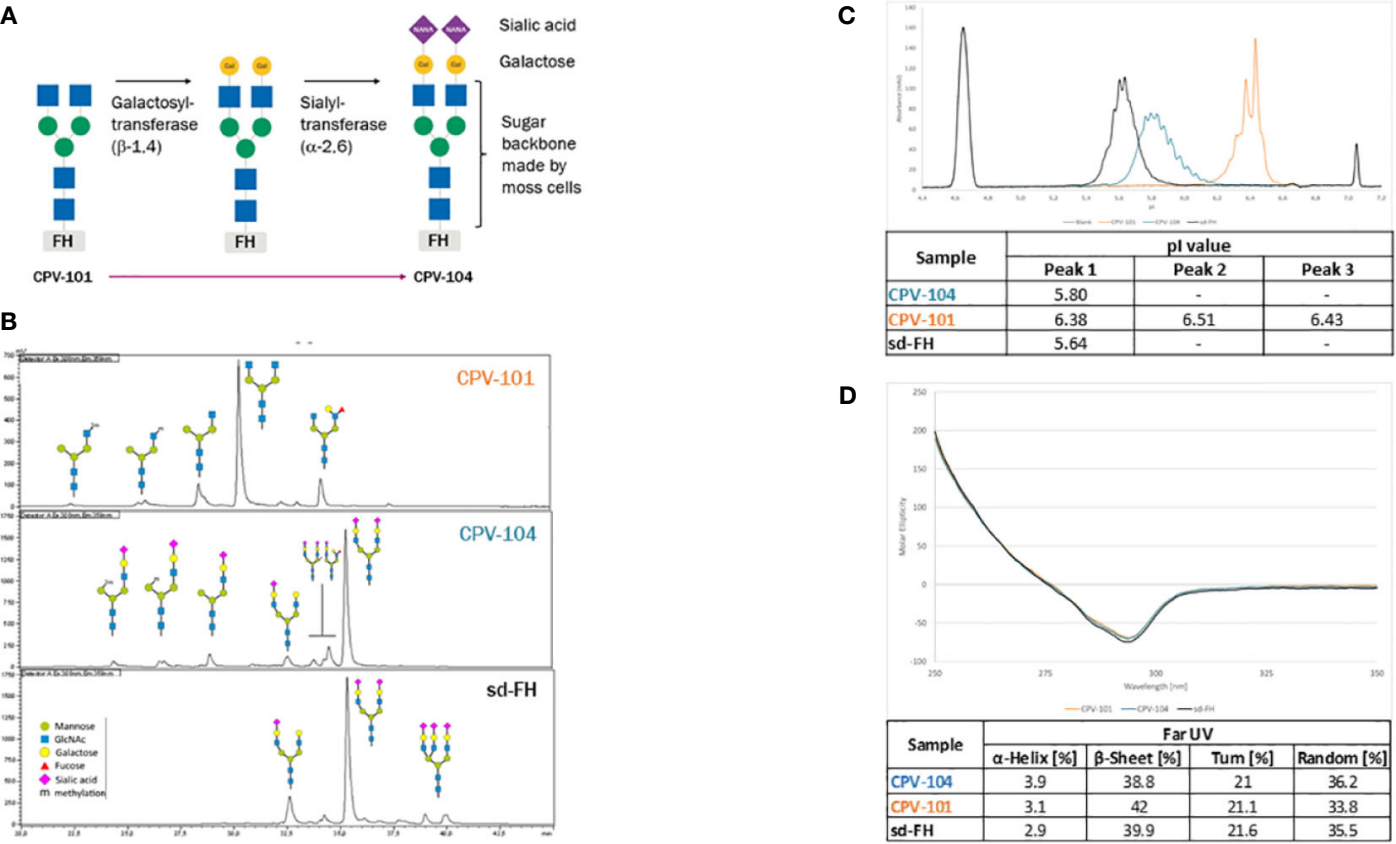
Structure and glycosylation pattern of three FH variants. **(A)** Enzymatic modification of CPV-101 to CPV-104 by the addition of galactose and sialic acid residues. **(B)** HILIC-FLD-HPLC analysis of moss-derived precursor CPV-101, glycan-optimized CPV-104 and sd-FH. **(C)** Overlay of electropherograms of the three FH variants (blue = CPV-104; orange = CPV-101; black = sd-FH) showing pI markers. **(D)** Overlay of CD spectra (near-UV range) of the three FH variants converted to molar ellipticity (blue = CPV-104; orange = CPV-101; black = sd-FH) and summary of the secondary structure fit (far-UV range).

The amino acid sequences of CPV-104 and CPV-101 match the sequence of human FH (UniProt ID: P08603) except position 62, where we selected the polymorphism 62I. Recombinant proteins were produced in moss suspension cultures cultivated in illuminated 500-L single-use stirred-tank reactors (Biostat STR500; Sartorius, Germany) as previously described (51). The clarified culture supernatant was concentrated by tangential flow filtration (30 kDa cut-off, regenerated cellulose) and loaded onto a FH affinity column (custom made, Repligen, USA) equilibrated with 20 mM Tris-HCl, 150 mM NaCl (pH 7.4). CPV-101 was eluted in a single step using 100 mM citric acid, 5% propylene glycol and 100 mM l-arginine (pH 3.0). The resulting pool was processed by two-step *in vitro* sialylation using β-1,4-galactosyltransferase and α-2,6-sialyltransferase (Roche Custom Biotech, Germany) according to the manufacturer’s protocol (**Figure 2A**). The resulting sialylated FH protein (CPV-104) was loaded onto a CaptoDeVirs column (Cytiva, Sweden) equilibrated with 20 mM Tris-HCl, 50 mM NaCl (pH 8.5) and eluted by increasing the NaCl concentration in a step gradient. The fraction containing CPV-104 was loaded onto a CaptoAdhere ImpRes column (Cytiva) equilibrated with 20 mM Tris-HCl, 500 mM NaCl (pH 8.5) and the pH was lowered to 5.0 with a washing step. CPV-104 was eluted by reducing the NaCl concentration in a step gradient. The fraction containing CPV-104 was formulated in 20 mM Tris-HCl (pH 8.5) containing 150 mM NaCl and 0.02% Tween-20 at a concentration of 10–20 mg/ml, and was stored at –80°C. Yields of an exemplary purification process are presented in **Supplementary Table S1**.

### N-glycan analysis by HILIC-HPLC-MS, cIEF and CD spectroscopy, and determination of the extinction coefficient by time-resolved amino acid analysis

See **Supplementary Material**.

### SPR analysis of FH-C3b binding

See **Supplementary Material**.

### Cofactor activity, decay acceleration activity, hemolysis assays and C3b binding assays

See **Supplementary Material** according to (51). The serum from patient aHUS1 was used in the hemolysis assay.

### Terminal complement complex ELISA

NUNC MaxiSorp 96-well plates were coated with 2.5 µg/well lipopolysaccharide (LPS; *Salmonella* serotype *enteritidis*; Sigma-Aldrich, USA) diluted in Dulbecco’s phosphate-buffered saline (DPBS; Gibco/Thermo Fisher Scientific, USA) overnight at 4°C. The plate was washed with wash buffer (DPBS + 0.05% Tween-20) and blocked with DPBS + 1% bovine serum albumin (BSA) at 37°C for 1 h. We diluted sd-FH, CPV101 and CPV104 in 100 µl GVB buffer containing 5 mM Mg-EGTA and 20% normal human serum (NHS). The samples were transferred to the plate and incubated at 37°C for 1 h. After washing, we added the C5b-9 antibody (clone aE11, sc-58935, 100 µg/ml, Santa Cruz Biotechnology, USA) diluted 1:2000 in wash buffer, and incubated for 1 h at room temperature. After washing, we added a horseradish peroxidase (HRP)-labeled anti-mouse antibody (NXA931, 2 mg/ml, Cytiva, USA) diluted 1:5000 in wash buffer, and incubated for 1 h at room temperature. The color reaction was started by adding TMB substrate to the wells for 10 min and stopped with H_2_SO_4_. We measured the absorbance at 450 nm using an EPOCH microplate reader (BioTek, USA). Data were normalized against untreated NHS.

### Bacteriolysis assay with *N. meningitidis* in NHS

*N. meningitidis* serotypes B and W were cultivated overnight and adjusted to OD_600 = _0.1 with 2×THY broth. The bacteria were then challenged with either pooled complement-active NHS (50%) or with heat-inactivated NHS (hiNHS) treated for 1 h at 56°C. NHS was supplemented with CPV-104 at concentrations of 4, 8, 15, 30, 60, 125 and 250 µg/ml, or with CPV-101, eculizumab or sd-FH as comparators. BSA was added at the same concentrations as a control. Bacteria were then incubated for 60 min at 37°C in a 5% CO_2_ atmosphere. After serum challenge, the samples were placed on ice to block complement activity. The bacteria were then serially diluted in THY broth and the diluted samples were transferred to blood agar plates. Following incubation at 37°C in a 5% CO_2_ atmosphere for 24 h, colonies were counted and presented as colony forming units (CFU). Each assay was carried out at least three times per serotype, starting from independent cultures.

### Inhibition of C5b-9 and C3 deposition on HMEC-1 cells

The ability of CPV-104 and sd-FH to inhibit C5b-9 formation on endothelial cells was assessed as previously described (59). Briefly, HMEC-1 cells were activated with 10 µM ADP, then incubated with either pooled NHS or primary aHUS serum (patients aHUS3–5, diluted 1:2 in HBSS + 0.5% BSA) in the presence or absence of CPV-104 (500 µg/ml), sd-FH (500 µg/ml) or pegcetacoplan (1 mg/ml) for 4 h at 37°C. The cells were then stained with an anti-human C5b-9 antibody (Calbiochem, 204903, 1mg/ml, 1h at room temperature, 1/200 final dilution) followed by a fluorochrome-conjugated secondary antibody (Jackson Immuno Research Laboratories, 1.5 mg/ml, 111-095-144, 1h at room temperature, 1/50 final dilution). Fifteen fields per sample were acquired and the areas with fluorescent staining were evaluated using the built-in automatic edge detection function of Image J. The highest and lowest values were discarded, and the mean was calculated on the remaining 13 fields.

For the examination of C3 deposits, HMEC-1 cells prepared as described above were incubated with either NHS or aHUS serum containing FH autoantibodies (patient aHUS2) for 4 h at 37°C. The cells were fixed in 4% paraformaldehyde (PFA) and stained with a rabbit anti-human C3c FITC-labeled antibody (F0201, 2.9 mg/ml, Dako, 1:300) to detect C3 and iC3b and DAPI to counterstain the nuclei.

### Platelet aggregates on HMEC-1 cells under flow conditions

To analyze the formation of platelet aggregates, HMEC-1 cells were prepared as described above and incubated for 3 h with pooled NHS or primary aHUS serum (patients aHUS6–8, diluted 1:2 in HBSS + 0.5% BSA) in the presence or absence of CPV-104 (500 µg/ml), sd-FH (500 µg/ml) or pegcetacoplan (1 mg/ml). The cells were then perfused in a flow chamber with heparinized whole blood from healthy subjects plus the fluorescent dye mepacrine to label platelets (60). After 3 min of perfusion, the endothelial cell monolayer was fixed in acetone. Fifteen images per sample were acquired, and areas containing thrombi were evaluated using Image J software. The highest and lowest values were discarded, and the mean was calculated on the remaining 13 fields.

### Inhibition of C3 deposition on the surface of PNH erythrocytes

The ability of CPV-104 and sd-FH to prevent C3 deposition on the surface of PNH erythrocytes was tested as previously described (61). Briefly, EDTA-treated whole blood was centrifuged for 5 min at 400 g and the erythrocytes were reconstituted in an equivalent amount of saline for three wash cycles. We then mixed 2 µl of erythrocytes with PBS alone or with 0.5 µM eculizumab combined with increasing concentrations of CPV-104 (0, 0.2, 0.5, 1, 2 and 5 µM) or 12 µM pegcetacoplan in a total volume of 10 µl. We then added 30 µl of ABO-matched, acid-activated (0.1 M HCl, diluted 1:10) NHS supplemented with 2 mM MgCl_2_ and incubated the cells for 24 h at 37°C. The cells were washed with PBS containing 2 mM EDTA and stained with antibodies against CD59 (376004, anti-human CD59 PE; 50 µg/ml, Biolegend, USA, 1:200) and C3c to detect C3 and iC3b (F0201, 2.9 mg/ml, Dako, USA, 1:25) before analysis by flow cytometry.

### *In vivo* experiments

### ^125^I radiolabeling to study exposure, tissue distribution and elimination of FH in wild-type mice

See **Supplementary Material**.

### Single-dose PK/PD study of CPV-104 and CPV-101 in FH-deficient (FH^-/-^) mice

FH-depleted mice were kindly provided by Matthew Pickering (Centre for Complement and Inflammation Research, Imperial College London, London, UK) and were bred externally by Charles River Laboratories. Male and female mice 8–16 weeks of age were used for the experiments. All procedures involving animals were conducted in accordance with the guide for the care and use of laboratory animals (published by the US National Institutes of Health and the German Animal Protection Code) and were approved by local authorities (Regierungspräsidium Freiburg G-21/111). We injected three mice each intravenously with CPV-104, CPV-101 or sd-FH at 40 mg/kg. At the indicated time points, blood samples were collected from the tail vein to determine serum FH and C3 concentrations. Four days after injection, the mice were humanely killed, and the kidneys were snap-frozen in liquid nitrogen for the analysis of glomerular C3 deposits. Serum samples from the mice were diluted 1:10.000 and the FH and C3 concentrations were determined using ELISA kits according to the manufacturers’ instructions (HK342 for FH, Hycult Biotech, Netherlands; ab157711 for C3, Abcam, UK). Glomerular C3 deposits were detected by immunofluorescence staining as previously described (51).

### Single-dose PK/PD study of CPV-104 and CPV-101 in cynomolgus monkeys

Studies with cynomolgus monkeys were carried out by Labcorp (Germany) and were approved by Landesamt für Natur, Umwelt und Verbraucherschutz Nordrhein-Westfalen, LANUV NRW, reference nos. 81-02.04.2016.A115 and 81-02.04.2021.A445). We injected 4–5-year-old male cynomolgus monkeys intravenously with a single dose (75 or 150 mg/kg, n = 2 per group) of CPV-104 or CPV-101. Serum samples were collected before treatment and at the indicated time points afterwards to measure the FH concentration and for complement analysis. The concentration of CPV-104 and CPV-101 in serum samples was measured using an FH ELISA kit (HK342, Hycult Biotech) according to the manufacturer’s instructions. No cross-activity with endogenous monkey FH was observed.

### Bacteriolysis assay with *E. coli*

Chemically competent *Escherichia coli* cells (18265017, Invitrogen, USA) were transformed by heat shock with plasmid pTRACER CMV2 (V88520, Invitrogen) conferring ampicillin resistance and plated overnight on lysogeny broth (LB) agar plates containing 100 µg/ml ampicillin. A single colony was picked and cultured overnight at 37°C in LB containing 100 µg/ml ampicillin. The bacterial suspension was diluted to OD_600 = _0.6 and 50 µl was then mixed with 150 µl cynomolgus monkey serum (treated with CPV-104 or CPV-101, or untreated) and incubated for 1 h at 37°C. The bacteria were plated on LB agar containing ampicillin as above and incubated overnight at 37°C. The colonies were counted and extrapolated to CFU/ml serum. Heat inactivated (56°C for 30 min) cynomolgus monkey serum was used as a control.

### Statistics

GraphPad Prism v8 (GraphPad Software, USA) was used for all statistical analysis and data visualization. IC50 values were calculated by x = log(x) data transformation, and curves were fitted using the nonlinear regression log (inhibitor) versus response (four parameters) setting. One-way analysis of variance (ANOVA) with Tukey’s or Dunnett’s multiple comparison test was used to determine statistical significance as indicated (***P < 0.001, **P < 0.01, *P < 0.05).

## Results

### Structure and glycosylation pattern of CPV-104

The polypeptide sequences of CPV-104 and CPV-101 are identical to native human FH (UniProt ID: P08603). Both recombinant proteins contain isoleucine at position 62, and tyrosine at position 402 (**Figure 1**; **Supplementary Figures S1A, B**). In contrast, sd-FH (used as a comparator in functional and structural assays) is a mixture of polymorphic variants purified from human serum pools. For example, the batch of sd-FH we analyzed contained ~38% isoleucine and ~62% valine at position 62 (**Supplementary Figure S1A**) as well as ~31% tyrosine and ~69% histidine at position 402 (**Supplementary Figures S1A, B**).

To generate CPV-104, the *N*-linked glycans of CPV-101 (mainly GlcNAc/GlcNAc) were enzymatically modified in two steps after purification to introduce sequential galactose and sialic acid (Neu5Ac) residues, such that CPV-104 has ~50% diantennary sialylated glycans like sd-FH, as confirmed by HILIC-FLD-MS. But CPV-104 in contrast to sd-FH, lacks triantennary sialylated glycans (**Figures 2A, B**, **Supplementary Table S2**). These differences in glycosylation between CPV-104 and the two other FH variants were additionally confirmed by capillary electrophoresis because the charged sialic acid residues influence the isoelectric point: pI_CPV-104_ = 5.8, pI_CPV-101_ = 6.4, and pI_sd-FH_ = 5.6 (**Figure 2C**). Notably, the molecular masses of CPV-104, sd-FH and CPV-101 were 151, 153 and 147 kDa, respectively (**Supplementary Figure S1C**). Removing the glycans with PNGase F equalized the molecular masses of all three FH variants, indicating that the different native masses were caused by distinct glycan profiles (**Supplementary Figure S1D**). No differences were observed in structure between the variants by circular dichroism (CD) spectroscopy (**Figure 2D**).

The extinction coefficients of CPV-101 and sd-FH were determined by time-resolved amino acid analysis, resulting in comparable values of 217,049 and 217,854 L/(mol*cm), respectively. For subsequent concentration measurements in further experiments, we used the value 1.58 [L/(g*cm)], re-calculated from 217,049 L/(mol*cm) with a molecular weight of 136,984.7 Da based on the amino acid composition of the FH sequence. Since protein extinction coefficient is determined by the amino acid composition and both CPV-variants have the same amino acid sequence the extinction coefficient for CPV-104 is equal to the one of CPV-101.

### Functional characterization of CPV-104 *in vitro*

**Figure 3:**
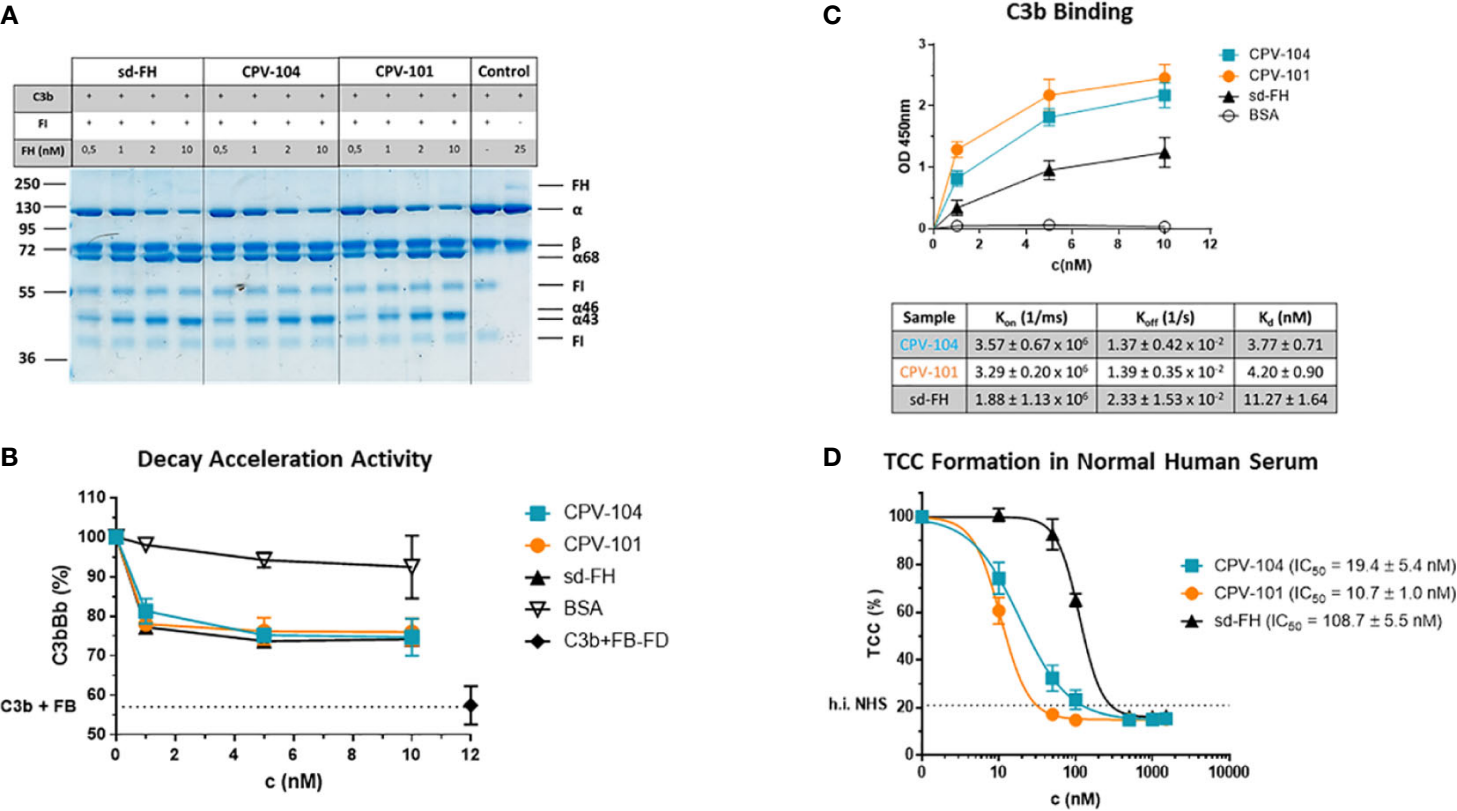
Functional activity of the FH variants *in vitro*. **(A)** The cofactor activity of moss-produced FH variants is similar to that of sd-FH. Increasing concentrations of CPV-104, CPV-101 or sd-FH were incubated with C3b and FI. C3b cleavage products (α’68, α’46 and α’43) were separated by SDS-PAGE and visualized by staining with Coomassie Brilliant Blue. Shown is a representative blot out of 3 independent experiments. **(B)** The decay acceleration activity of CPV-104 and CPV-101 is identical to that of sd-FH. C3 convertase was prepared by incubating C3b with FD and FB. The convertase was then incubated with increasing concentrations (1 nM, 5 nM, 10 nM) of CPV-104, CPV-101, sd-FH or BSA and leftover Bb fragments were measured by ELISA. Results are shown as mean ± SD of at least n = 3 independent experiments. **(C)** CPV-104 and CPV-101 bind with greater affinity than sd-FH to C3b. Wells were coated with C3b and then incubated with different concentrations (1 nM, 5 nM, 10 nM) of sd-FH, CPV-101, CPV-104 or BSA. Results are shown as mean ± SD of at least n = 3 independent experiments. The affinity of C3b binding was validated by SPR spectroscopy (data summarized in table show means ± SD from 3 independent measurements). **(D)** CPV-104 and CPV-101 inhibit TCC formation at significantly lower concentrations than sd-FH. Mg-EGTA-treated NHS was incubated with increasing concentrations of CPV-104, CPV-101 or sd-FH on LPS-coated microtiter plates to activate the alternative pathway. The quantity of C5b9 complexes was determined by ELISA. Data were normalized against untreated NHS samples. IC_50_ values are shown in nM. Results are shown as mean ± SD of at least n = 3 independent experiments.

CPV-104 was characterized *in vitro* to confirm its functional activity. CPV-104, CPV-101 and sd-FH showed comparable activity in C3b cleavage (**Figure 3A**; **Supplementary Figure S2**) and decay acceleration (**Figure 3B**) assays. However, moss-produced CPV-104 and CPV-101 bound with higher affinity to C3b than sd-FH, as determined by enzyme-linked immunoassays (ELISAs) and surface plasmon resonance (SPR) spectroscopy (3.77×10^-9^ vs 11.27×10^-9^) (**Figure 3C**). In addition, CPV-104 and CPV-101 inhibited the alternative pathway (AP) in NHS 10-fold more efficiently than sd-FH, with IC_50_ values of 19 nM for CPV-104, 11 nM for CPV-101, and 109 nM for sd-FH (**Figure 3D**).

**Figure 4:**
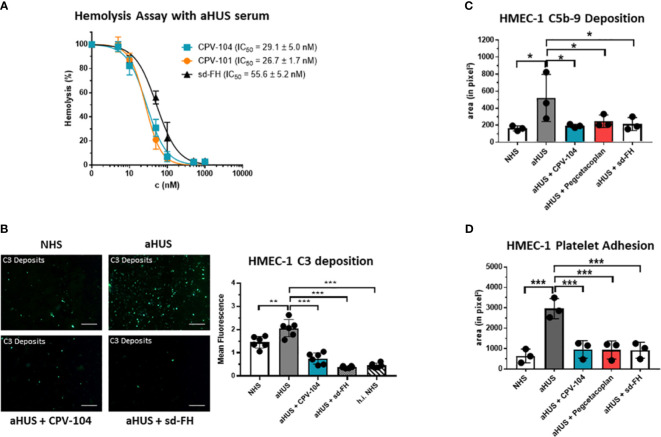
CPV-104 restores proper complement activity in the serum of aHUS patients. **(A)** CPV-104 and CPV-101 block hemolysis in aHUS serum and act at lower concentrations than sd FH. Data were normalized against untreated aHUS serum. IC50 values are shown in nM. Results are shown as mean ± SD of at least n = 3 independent experiments. **(B)** CPV-104 inhibits pathogenic C3 deposition on endothelial cells when added to the serum of aHUS patients. Untreated aHUS serum causes the abundant deposition of C3 molecules on HMEC-1 cells, which was prevented by adding either 1 µM (~155 µg/ml) CPV-104 or sd-FH to the serum (h.i.NHS – heat-inactivated NHS). Shown is a representative experiment out of 3 independent experiments using aHUS2 serum. Mean fluorescence intensity of 6 fields was analyzed for each sample. Results are shown as mean ± SD. Scale Bar = 50 µm. One-way ANOVA using Turkeys’s multiple comparison test (**P<0.01; ^***^P<0.001). **(C)** CPV-104 inhibits C5b-9 deposition on endothelial cells when added to the serum of aHUS patients (n=3). HMEC-1 cells were activated with ADP and incubated with normal human serum or serum from three aHUS patients treated with or without CPV-104, pegcetacoplan or sd-FH. Untreated aHUS serum causes the abundant deposition of C5b-9 on HMEC-1 cells, which was prevented by the addition of CPV-104, pegcetacoplan or sd-FH to the serum. Results shown are the means ± SD of 3 independent experiments using 3 different aHUS sera (aHUS3, 4, 5). One-way ANOVA using Turkeys’s multiple comparison test (*P<0.05). **(D)** Pre-exposure to untreated aHUS serum results in the formation of large platelet aggregates on HMEC-1 cells upon perfusion with normal whole blood. Results shown are the means ± SD of 3 independent experiments using 3 different aHUS sera (aHUS6, 7, 8). Results are shown as mean ± SD. One-way ANOVA using Turkeys’s multiple comparison test (***P<0.001).

FH distinguishes between self and non-self surfaces to control complement activation on intact host cells. We therefore conducted a hemolysis assay using sheep erythrocytes in sera from aHUS patients. CPV-104 and the other FH variants protected the erythrocytes from complement-mediated lysis, although the potency of CPV-104 104 (IC_50_ = 29 nM) and CPV-101 (IC_50_ = 27 nM) was almost two fold higher than that of sd-FH (IC_50_ = 56 nM) (**Figure 4A**). Having shown that the post-production sialylation does not compromise protein functionality and that CPV-104 retains the same functionality as CPV-101, further *in vitro* complement assays focused exclusively on assessing CPV-104 efficacy.

In aHUS patients, aberrant activation of the AP causes endothelial cell injury, exposing the subendothelium and leading to the common features of thrombotic microangiopathy. To determine whether CPV-104 prevents pathologic complement deposition on activated human microvascular endothelial cells (HMEC-1), ADP-stimulated HMEC-1 cells were challenged with serum from three primary aHUS patients (aHUS3-5) and C5b-9 deposits were detected by fluorescence staining. A significant increase in the quantity of cell-surface C5b-9 was observed following incubation with aHUS patient sera compared to NHS (**Figure 4C**). This effect was ameliorated when CPV-104 was added to the aHUS sera. Related effects were observed for aHUS sera supplemented with sd-FH or pegcetacoplan. Similarly, the quantity of C3 deposits was significantly higher with serum from an aHUS patient containing FH autoantibodies (aHUS2) compared to NHS (**Figure 4B**). Again CPV-104 and sd-FH ameliorated this effect. In contrast, incubation with eculizumab did not prevent the formation of C3 deposits on HMEC-1 cells.

In aHUS, complement activation causes the loss of endothelial thromboresistance resulting in microvascular thrombosis. We investigated the ability of CPV-104 to prevent the formation of platelet aggregates on microvascular endothelial cells. In agreement with earlier results (60), we observed an average six-fold increase in the cell surface area covered by platelet aggregates in ADP-activated HMEC-1 cells pre-exposed to serum collected from primary aHUS patients during the acute phase, and then perfused with heparinized whole blood, compared to cells exposed to pooled control serum (**Figure 4D**). The addition of CPV-104, pegcetacoplan or sd-FH to the serum led to a significant reduction in the cell surface area covered by platelet aggregates compared to untreated serum (**Figure 4D**). Notably, CPV-104 inhibited thrombus formation induced by aHUS serum with an efficiency comparable to that of sd-FH at the same concentration (**Figure 4D**).

**Figure 5:**
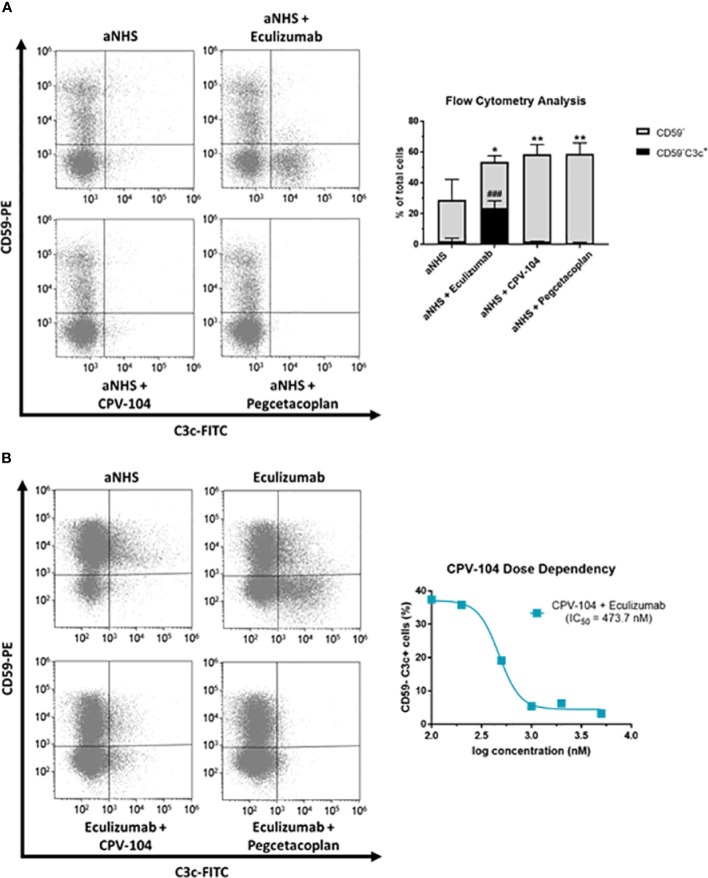
CPV-104 protects PNH erythrocytes from complement-mediated opsonization. **(A)** Left – PNH erythrocytes are characterized by the absence of the cell surface complement regulator CD59, rendering them susceptible to complement-mediated lysis when exposed to ABO-matched acidified serum. Eculizumab prevents the lysis of PNH erythrocytes but leaves C3 deposits on the surface of CD59^–^ erythrocytes, making them targets for extravascular hemolysis. Right – Summary and analysis of flow cytometry data from three PNH patients. Complement activation using acidified normal human serum (aNHS) leads to the significant lysis of CD59^–^ erythrocytes. Treatment with eculizumab prevents the lysis of CD59^–^ erythrocytes, but a significant number of C3 deposits can be detected (CD59^–^C3c^+^). In contrast, treatment of PNH erythrocytes with CPV-104 or pegcetacoplan prevents the lysis of erythrocytes and pathologic complement deposition. Results are shown as mean ± SD from 3 independent experiments. One-way ANOVA using Dunnett’s multiple comparison test against aNHS for CD59^–^ and CD59^–^C3c^+^ cells, respectively (*P<0.05; **P<0.01; ^###^P<0.001). **(B)** Erythrocytes from PNH patients were incubated with aNHS alone, with eculizumab, or with a combination of eculizumab and the C3 inhibitor pegcetacoplan or CPV-104 for 24 h. Erythrocytes were subsequently stained for CD59 and C3c, and the percentages of positively and negatively stained cells were analyzed by flow cytometry. Left: Treatment of erythrocytes with eculizumab plus pegcetacoplan or CPV-104 effectively prevents pathological C3 deposition on PNH erythrocytes. Right: Using the same experimental setup, erythrocytes from PNH patients were treated with eculizumab in combination with increasing concentrations of CPV-104 and the number of C3c^+^ PNH erythrocytes analyzed by flow cytometry. At concentrations above 1000 nM, pathologic C3 deposition on PNH erythrocytes could be completely prevented by CPV-104.

PNH patients lack surface complement regulators CD55 and CD59 on the progeny of GPI-deficient stem cells, which leads to intravascular hemolysis. Furthermore, terminal complement inhibition inevitably leads to the formation of C3 deposits on erythrocytes, resulting in opsonization and extravascular destruction by macrophages in the liver and spleen. Many PNH patients treated with eculizumab therefore still suffer from anemia. Proximal complement inhibitors and FH have the advantage of acting at the C3 level and can prevent the C3-mediated opsonization of erythrocytes and subsequent extravascular hemolysis. To test this hypothesis, erythrocytes from PNH patients (PNH1–PNH4) were treated with ABO-matched sera containing CPV-104, sd-FH, pegcetacoplan, or eculizumab. Complement was activated with HCl (61, 62). After 24 h, the reaction was stopped and complement deposits (C3) on the surface of erythrocytes were analyzed by flow cytometry. All the tested inhibitors led to a significant increase in the abundance of CD59^–^ erythrocytes, but eculizumab treatment led to a significant increase in the number of C3 deposits on erythrocytes whereas CPV-104 and pegcetacoplan almost completely prevented the formation of C3 deposits (**Figure 5A**). In a similar experiment, we assessed the dose-response relationship of CPV-104 regarding C3 deposition on PNH erythrocytes. Erythrocytes were treated with eculizumab in combination with increasing concentrations of CPV-104. CPV-104 exhibited a dose-dependent reduction in C3 deposits on PNH erythrocytes, yielding a calculated IC50 of approximately 473.7 nM (**Figure 5B**).

### CPV-104 maintains the bactericidal activity of human serum towards pathogenic *N. meningitidis*

**Figure 6:**
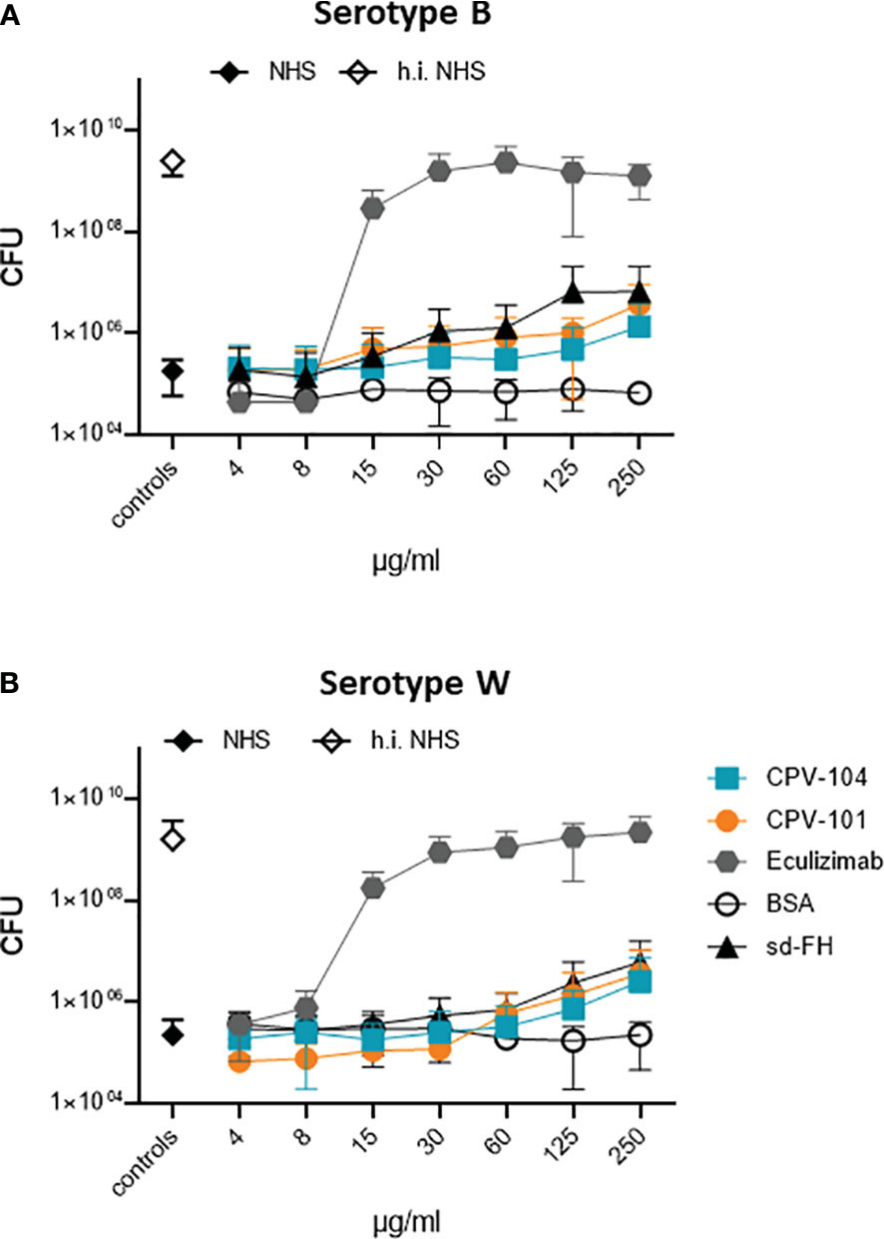
In presence of CPV-104 the bactericidal activity of human serum toward pathogenic *Neisseria meningitidis*. *N. meningitidis* remains intact. **(A)** serotype B and **(B)** serotype W when challenged with complement-active NHS (50%) are damaged and do not grow (black diamond). When complement is inactivated by heat treatment, bacteria survive resulting in bacterial growth (white diamond). In the presence of CPV-104 (blue squares) complement-mediated damage is observed. Similar effects are observed for CPV-101 (orange circles) and sd-FH (black triangles). In contrast, eculizumab (gray hexagon) blocks complement activity thus resulting in bacterial growth. Results are shown as mean ± SD of at least n = 3 independent experiments.

To determine whether CPV-104 interferes with the bactericidal activity of complement, we tested its effect on the TCC-mediated killing of pathogenic *N. meninigitidis* serotypes B and W. The two strains were challenged with complement-active NHS (50%) and bacterial survival was scored by colony counting. Bacteria were damaged by complement-active NHS, but not by hiNHS (**Figure 6**). CPV-104 at concentrations ≤ 250 µg/ml did not strongly affect complement-mediated bacteriolysis (**Figures 6A, B**). CPV-101 and sd-FH showed similar effects. In contrast, the TCC inhibitor eculizumab blocked complement mediated bacteriolysis, resulting in the growth of both *N. meningitides* serotypes (**Figures 6A, B**). The highest concentration used in this assay (250 µg/ml) would represent a total of maximum 1000 µg/ml FH in 100% normal human serum (300-500 µg/ml average FH concentration in NHS + 500 µg/ml CPV-104, CPV-101 or sd-FH).

### Characterization of CPV-104 *in vivo*: pharmacokinetics and organ biodistribution in wild-type mice

**Figure 7:**
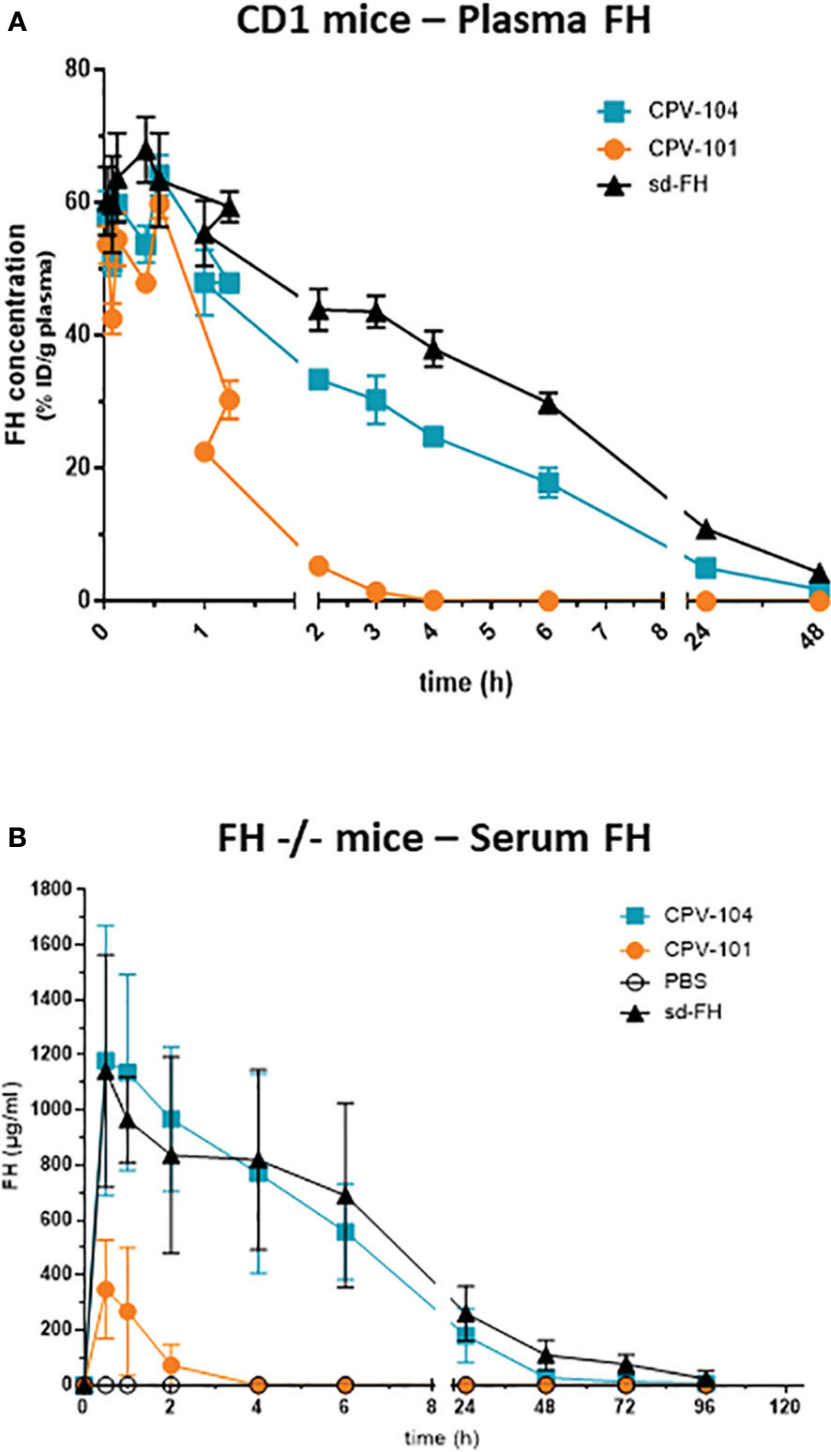
CPV-104 shows a favorable pharmacokinetic profile *in vivo*. Pharmacokinetic profiles following the intravenous injection of **(A)** ^125^I-labeled FH variants into wild-type (CD-1) mice and **(B)** FH variants into FH^–/–^ mice. Results are shown as mean ± SD from n = 3 separate animals.

We assessed the pharmacokinetic profile and biodistribution of CPV-104, sd-FH and CPV-101 in wild-type mice to determine whether glycan sialylation extended the half-life in circulation as anticipated. All three FH variants were radiolabeled with ^125^I and injected intravenously into wild-type CD-1 mice. Radioactivity in the blood was measured over a period of 48 h. As expected, CPV-104 showed a significantly longer serum half-life (~2.7 h) than CPV-101. The latter was rapidly cleared from the blood, with ~50% of the radioactivity eliminated within 30 min, corresponding to a serum half-life of ~35 min. However, CPV-104 still cleared more rapidly than sd-FH with a serum half-life of ~5.35 h (**Figure 7A**).

The organ distribution of the FH variants was determined 30 min after injection (**Supplementary Figure S3**). The concentration of CPV-101 in the liver was much higher compared to the other variants. Both CPV-104 and CPV-101 were also present at double the concentration of sd-FH in the kidneys. Elimination of all FH variants from systemic circulation was predominantly achieved by renal clearance (≥ 80% of the injected dose was found in the urine), whereas the liver and spleen appeared to be involved in protein metabolism.

### Characterization of CPV-104 *in vivo*: pharmacokinetics and efficacy in FH^–/–^ mice

Next, we investigated whether the prolonged serum half-life of CPV-104 could improve clinical parameters in a mouse model of C3G. FH knockout mice (FH^–/–^) are characterized by a constitutively activated AP, which leads to low serum C3 levels and pronounced C3 deposits in the kidneys. In agreement with the results observed in wild-type CD-1 mice, the half-life of CPV-104 was greatly extended compared to CPV-101 in the FH^–/–^ mice. The peak concentration (C_max_) and serum half-life of CPV-104 did not differ significantly from those of sd-FH (**Figure 7B**).

**Figure 8:**
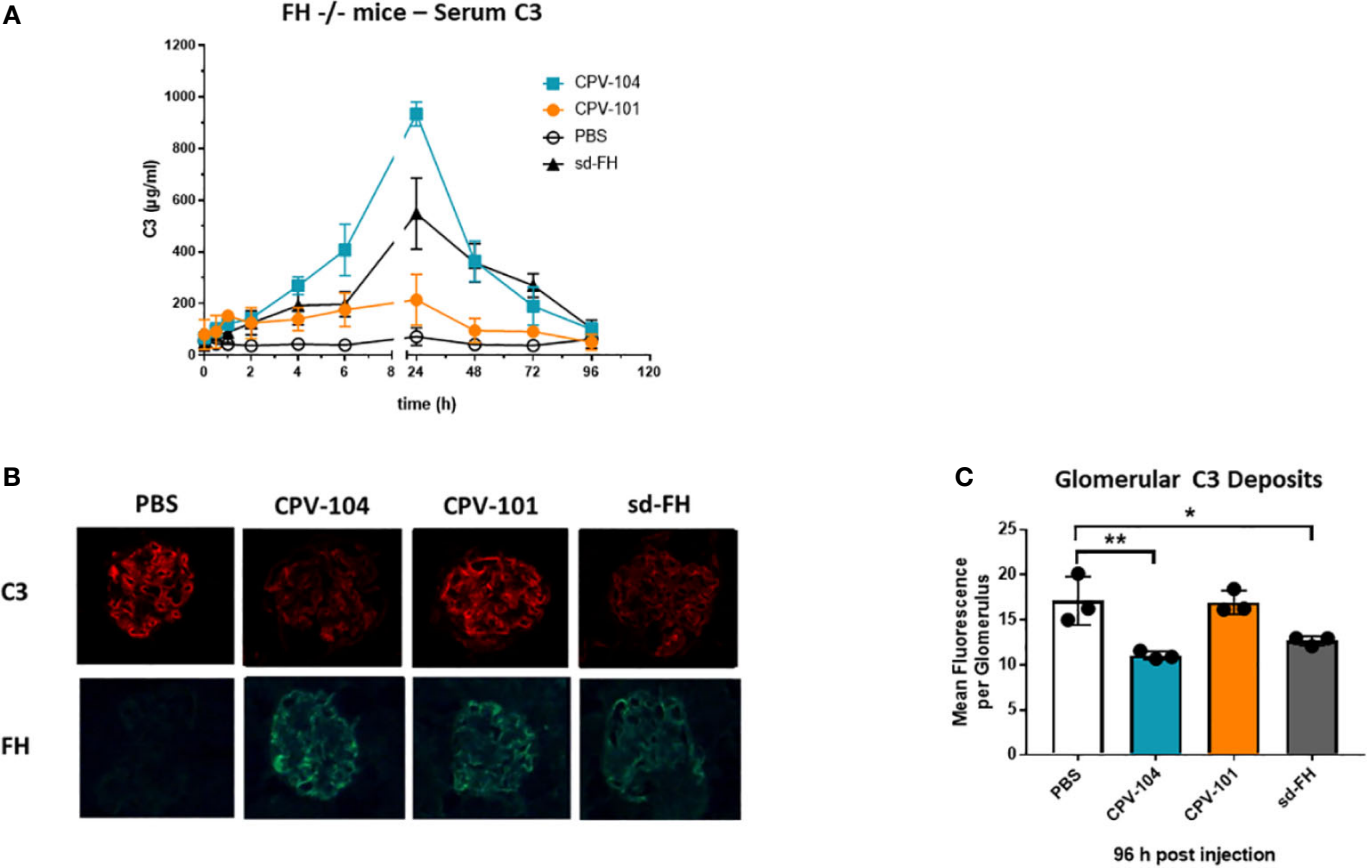
CPV-104 can ameliorate pathogenic conditions in FH^–/–^ mice. **(A)** In FH^–/–^ mice, the injection of CPV-104 and sd-FH led to comparable normalization of serum C3 levels. Results are shown as mean ± SD from n = 3 separate animals. **(B, C)** In FH^–/–^ mice, the analysis of complement C3 deposits 4 days after the injection of FH variants revealed a significant reduction in glomerular C3 deposits in response to CPV-104, comparable to sd-FH. All three FH variants were still detected 4 days post-injection in the glomeruli of FH^–/–^ mice (FH-specific staining). Results are shown as mean ± SD from at least 30 glomeruli each from n = 3 separate animals. One-way ANOVA using Turkeys’s multiple comparison test (*P<0.05; **P<0.01).

Serum C3 levels in the FH^–/–^ mice were normalized 24 h after the injection of CPV-104 or sd-FH, with both variants achieving comparable effects, whereas the injection of CPV-101 had a much weaker effect (**Figure 8A**). The mice were humanely killed after 96 h and glomerular C3 deposits were analyzed by fluorescence microscopy (**Figure 8B**). In addition, FH binding to glomeruli was visualized using FH-specific antibodies. CPV-101, CPV-104 and sd-FH were all detected in glomeruli 96 h after injection, but only the treatments with CPV-104 and sd-FH led to a significant reduction in the intensity of glomerular C3 deposits (**Figure 8C**).

### Pharmacokinetics and efficacy in non-human primates

**Figure 9:**
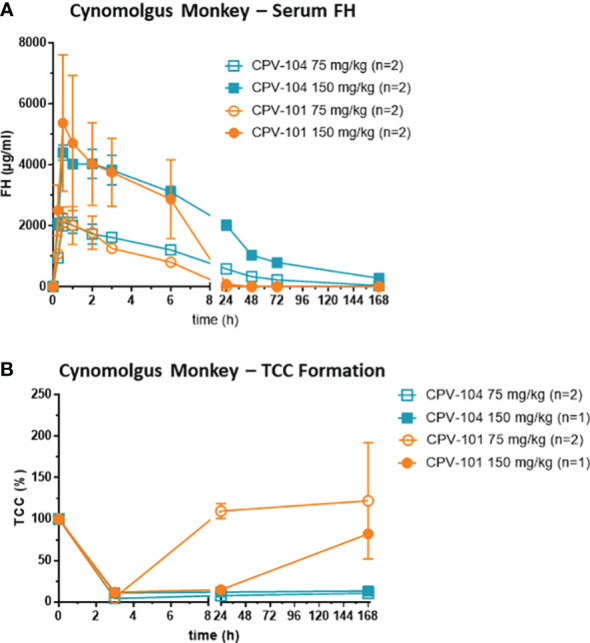
CPV-104 pharmacokinetic profiles and efficacy in cynomolgus monkey are superior to CPV-101. **(A)** Pharmacokinetic profiles of CPV-104 and CPV-101 following intravenous injection in cynomolgus monkeys. Results are shown as mean ± SD from n = 2 separate animals. **(B)** At supraphysiological levels, CPV-104 inhibits LPS-triggered TCC formation in monkey serum. The effect achieved with CPV-104 lasted significantly longer than that of CPV-101. Data are means ± standard deviations from n = 2 separate animals for the dose group 75 mg/kg and values from single animals are presented for the dose group 150 mg/kg. Two animals were excluded from the analysis due to significantly lower TCC activity of predose serum samples compared to the rest of the cohort.

Finally, we assessed the therapeutic potential of CPV-104 using cynomolgus monkeys, a preclinical model that is closer to human physiology than mice. Male monkeys (n = 2 per group) were injected intravenously with 75 mg/kg or 150 mg/kg of CPV-104 or CPV-101, and serum concentrations of the compounds were monitored for 7 days. The peak concentrations (C_max_) of CPV-104 and CPV-101 were recorded 30 min post-injection (**Figure 9A**) and were dose-dependent (~2 mg/ml for the 75 mg/kg dose and ~5 mg/ml for the 150 mg/kg dose). Serum concentrations of CPV-104 remained high until 24 h post-injection and CPV-104 was still detectable in the serum on day 7. In contrast, CPV-101 serum concentrations decreased rapidly and were undetectable 24 h post-injection in both dose groups. The serum half-life of CPV-104 (~50 h) was ~11-fold longer than that of CPV-101 (~4.6 h).

To evaluate the efficacy of CPV-104, monkey serum was collected over several time points (0, 3 and 168 h post injection) and its ability to form TCCs after activation with LPS was determined. In serum of all monkeys, TCC formation was inhibited 3 h post-injection, indicating the supraphysiological FH-mediated suppression of complement activation (**Figure 9B**). Treatment with CPV-104 led to a pronounced suppression of TCC activity lasting up to 7 days regardless of the dose. In comparison, TCC formation in the CPV-101 75 mg/kg group normalized already after 24 h, whereas complement suppression in the CPV-101 150 mg/kg group could still be observed 24 h post-injection (**Figure 9B**).

Despite the suppression of complement activation *in vitro*, FH can differentiate between self-surfaces and foreign surfaces, and controls complement action on intact host cells but still allows complement activation on the surface of infectious microbes. We therefore assessed how CPV-104 affects the ability of serum from monkeys to destroy *E. coli* cells. Monkey serum sampled before and 3 h after CPV-104 treatment was incubated with ampicillin-resistant *E. coli* and the number of colonies was counted after overnight incubation on agar plates. No colonies were formed when the bacteria were incubated with monkey serum before and after treatment with CPV-104, indicating that complement activity against *E. coli* was retained and was not affected by CPV-104 (**Supplementary Figure S4**). In contrast, *E. coli* cells remained intact when treated with heat-inactivated monkey serum.

## Discussion

We here present a follow-up to our previous study demonstrating that recombinant FH produced in moss (CV-101) is suitable as a replacement therapy for the treatment of complement-associated disorders (51). The new FH version discussed herein (CPV-104) has a modified glycosylation pattern featuring terminal sialic acid residues that improve the half-life and biological activity of this prospective therapeutic drug.

The detailed biochemical characterization of CPV-104 revealed no significant structural changes compared to sd-FH (**Figure 2D**). Minor differences in molecular mass and pI between the two FH variants reflect remaining distinctions in the glycosylation pattern (i.e., sd-FH carries a small number of triantennary sialylated glycans that are not present on CPV-104) (**Figure 2**; **Supplementary Table S2**). The slightly lower degree of sialyation in CPV-104 compared to sd-FH also probably explains its shorter half-life *in vivo* (**Figures 7A, B**). However, glycosylation is not thought to influence the functional activity of FH (30), and our *in vitro* complement functionality assays confirmed that CPV-104 retains all the complement regulatory functions of CPV-101 and sd-FH, and has the same level of cofactor and decay acceleration activity (**Figures 3A, B**).

CPV-104 and CPV-101 bound with significantly higher affinity than sd-FH to C3b (**Figures 3C, D**). Furthermore, hemolytic and TCC assays revealed that CPV-104 and CPV-101 inhibited complement activation at lower concentrations than sd-FH (**Figure 4**). The three factor H variants differ structurally in their glycosylation. However, as previously described, the glycosylation pattern does not affect C3b binding (30). This observation was confirmed by SPR Biacore measurements of CPV-101 and CPV-104 variants with the same polymorphism (Ile62/Tyr402). Despite differences in glycosylation (**Figure 2**), almost identical binding affinities were measured (**Figure 3C**).

Therefore, the observed differences in binding affinities between CPV-101, CPV-104 and sd-FH can be attributed to the differences in amino acid sequence at polymorphic positions. CPV-101 and CPV-104 are produced recombinantly, resulting in a strictly defined amino acid sequence and a homogenous preparation of FH molecules with identical amino acids at polymorphic positions.

In contrast, sd-FH (used as a comparator in functional assays) is a mixture of different variants purified from human serum pools (**Supplementary Figures S1A, B**). The amino acid at position 62 has a strong influence on the affinity of FH for C3b (63). The Ile62 variant has greater affinity for C3b and is ~20% more active in hemolytic assays than the Val62 variant. The differences between the moss-produced FH variants and sd-FH were more pronounced, with the former showing a two-fold increase in inhibition of hemolytic activity, which may reflect the unknown cumulative impact of additional polymorphisms in the sd-FH preparation. For example, we also expressed and purified a Val62/His402 variant during the development of CPV-101, and its C3b binding affinity was found to be comparable to that of sd-FH, but 1.5-fold lower than that of the CPV-101 Ile62/Tyr402 variant (**Supplementary Table S3**). Stronger binding properties of defined polymorphic variants could also influence interactions with cellular matrices (64). Accordingly, our radiolabeling experiments demonstrated that both CPV-104 and CPV-101 accumulated preferentially in the kidneys of wild-type mice compared to sd-FH (**Supplementary Figure S3**). The anticipated predominant targeting of CPV-101 to the liver probably reflects clearance by mannose-receptor C-type 1, which is strongly expressed on liver sinoidal endothelial cells and is known to accept GlcNAc-tipped glycans as ligands (65). The excretion data for all three variants were in overall good agreement with the previously published results for sd-FH (66).

As anticipated, CPV-104 has a much longer half-life *in vivo* than CPV-101, ~5-fold longer in mice and ~11-fold longer in cynomolgus monkeys (**Figures 7**, **9A**). This increased bioavailability leads to higher biological activity. For example, the ability of CPV-104 to increase serum C3 levels and reduce the number of C3 deposits in the kidneys of FH^–/–^ mice was comparable to sd-FH (**Figures 8A, C**). Similarly, complement inhibition (assessed by TCC formation) lasted up to 7 days after the injection of CPV-104 in cynomolgus monkeys whereas inhibition caused by the same dose of CPV-101 started to wear off after 24 h (**Figure 9B**). However, interpretation of the effects in cynomolgus monkeys is complicated by the lack of endogenous pharmacodynamic markers (such as low serum C3 levels and glomerular C3 deposits) and the fact that human FH shows greater efficacy than endogenous cynomolgus monkey FH (67). Furthermore, it is of note that the bactericidal activity against *E. coli* remained intact in CPV-104 treated cynomolgus monkeys despite the complete suppression of TCC formation on LPS-coated plates. One plausible explanation for this observation might lie in the nature of the employed assays. The TCC ELISA assesses alternative pathway activation exclusively within a controlled environment using only LPS as the triggering agent. In contrast, in our bactericidal assay using *E. coli* the sera from CPV-104 treated monkeys are exposed to a living pathogen, whose complex cell surface can cause complement activation in various ways, i.e. additional activation through the classical and lectin pathway cannot be fully excluded.

Considering the efficacy of CPV-104 in FH^–/–^ mice, an established C3G disease model, the almost complete elimination of C3 deposits (the main drivers of kidney pathology in C3G) after 96 h is an important achievement. This effect has been described following the treatment of FH^–/–^ mice with sd-FH (68) and the treatment of FH-deficient pigs with isolated porcine FH (69). Interestingly, the positive effect on C3 deposits can be perceived as soon as 24 h after injection, indicating that the FH effect sets in quickly (51, 68). The treatment of C3G patients with CPV-104 may therefore result in a rapid therapeutic effect. At present, due to the limited species specificity of available complement inhibitors such as pegcetacoplan, which primarily targets complement of humans and non-human primates (70, 71), there are no comparable data that allow us to assess the rate at which C3 deposits are removed from the kidneys in the FH^–/–^ mouse model. However, given the mode of action of C3 inhibitors, we assume that they do not promote the degradation of glomerular C3 deposits. In broader terms, it should be noted that the FH^–/–^ mouse model has limitations, as it differs in several key aspects from human C3G. FH^–/–^ mice exhibit a total absence of FH, a condition rarely found in human patients. Furthermore, the FH^–/–^ mice do not possess C3Nef antibodies, which are frequently present in human C3G cases. Additionally, rare gain-of-function mutations in C3 and FB also exist as causative factors in human C3G. All these factors could affect the ability of CPV-104 to effectively regulate complement. Nonetheless, it has been previously demonstrated that sd-FH is capable of overcoming the effects of C3Nef antibodies (72). Therefore, it can be assumed that increased (supraphysiological) FH levels achieved with CPV-104 administration would be able to attenuate an overactive complement system even under these conditions. As additional clinical data will become available in the future, further research will elucidate these aspects.

Currently, the focus of complement therapeutics is the systemic blockade of complement activity at different steps of the cascade. This strategy is successful when the main pathological drivers are the production of excess anaphylatoxins or TCCs (73). However, AP dysregulation is often more subtle and is probably caused by several factors that shift the balance of the complement system into a proactive state that ultimately reaches a kinetic tipping point (74). For example, no mutations in complement genes have been detected in 40–60% of aHUS patients, and even in families with genetic defects the penetrance of the disease is often incomplete (75, 76). This indicates that other factors drive complement dysregulation, some of which may be transient, such as injuries or infections (74). In these cases, CPV-104 would be advantageous as a natural regulator of the AP that would rebalance the complement system, as supported by its efficacy in limiting C5b-9 formation and platelet aggregates on HMEC-1 cells induced by aHUS serum *in vitro* (**Figure 4**).

Complement regulation as a main or adjunct therapy is increasingly recognized beyond the primarily complement-driven diseases such as aHUS, C3G and PNH (77, 78). AMD is the most common cause of vision loss in the elderly, and certain FH polymorphisms are strongly associated with this disease (79, 80). In rheumatoid arthritis, complement activation is involved in all stages of pathogenesis (81). Furthermore, the physiological role of the complement system far exceeds the opsonization and lysis of pathogens. Especially in neurobiology, it is now understood that the complement system is deeply involved with the development of the brain and central nervous system (82). An overactive complement system can cause severe brain injury, but anaphylatoxins are involved in neuroprotection and regeneration (83–85). Systemic complement inhibition is therefore likely to cause detrimental side effects, whereas regulators such as FH can prevent pathological complement activation while allowing physiological complement reactions. This is evident from our bacteriolysis assays, where even high concentrations of FH did not interfere with bacterial growth inhibition and lysis (**Figure 6**). Treatment with CPV-104 could therefore reduce or even prevent the risk of potentially life-threatening infections that are associated with other complement inhibitors.

The availability of substantial amounts of functional, full-length recombinant FH will facilitate its use in many clinical areas. There is mounting evidence that FH regulates diverse processes by interacting with proteins unrelated to the complement pathway (77). For example, FH may regulate or inhibit the mTOR pathway (86), and binds to apolipoprotein E (apoE), a major protein related to Alzheimer’s disease (87–89). It also interacts with C-reactive protein and malondialdehyde-modified proteins (87, 90). The precise role of FH in these interactions and in cell based chronic inflammation is not yet fully understood, but they all appear to support a role for FH as an anti-inflammatory mediator (91). A reliable source of recombinant FH could be further used to coat biomaterials and nanoparticles to protect their surfaces from opsonization and complement attack (92–95). FH could also be used to prevent ischemic reperfusion injuries during organ transplants (96).

In conclusion, our moss-based expression platform was used to produce CPV-104, a full-length recombinant FH with optimized glycans, in amounts sufficient for systemic therapy. Preclinical experiments showed that the pharmacokinetic and pharmacodynamic profile was comparable to that of sd-FH. The mode of action, based on the regulation and fine-tuning of an unbalanced complement system, offers an alternative therapeutic approach to available complement inhibitors. Based on these promising results we are now planning phase 1 clinical trials with CPV-104 in C3G patients.

## Data availability statement

The original contributions presented in the study are included in the article/**Supplementary Material**, further inquiries can be directed to the corresponding author/s.

## Ethics statement

The studies involving humans were approved by Ethics Committee of Freiburg (368/10) and Ethics Committee of Azienda Socio-Sanitaria Territoriale Papa Giovanni XXIII. The studies were conducted in accordance with the local legislation and institutional requirements. Written informed consent for participation in this study was provided by the participants’ legal guardians/next of kin. The animal studies were approved by Regierungspräsidium Freiburg (35-9185.81/G-21/111), Landesamt für Natur, Umwelt und Verbraucherschutz Nordrhein-Westfalen (81-02.04.2016.A115 and 81-02.04.2021.A445) and The French Ministry of Higher Education and Research (Apafis-27745, C2EA-06). The studies were conducted in accordance with the local legislation and institutional requirements. Written informed consent was obtained from the owners for the participation of their animals in this study.

## Author contributions

TT: Conceptualization, Formal analysis, Investigation, Methodology, Project administration, Visualization, Writing – original draft. SK: Formal analysis, Investigation, Validation, Visualization, Writing – review & editing. AB: Conceptualization, Data curation, Project administration, Writing – review & editing. CS: Methodology, Validation, Writing – review & editing. AH: Formal analysis, Investigation, Methodology, Resources, Validation, Visualization, Writing – review & editing. MN: Conceptualization, Data curation, Writing – review & editing. SG: Investigation, Writing – review & editing. SA: Data curation, Formal Analysis, Writing – review & editing. AS: Conceptualization, Writing – review & editing. JP: Resources, Writing – review & editing. PFZ: Conceptualization, Investigation, Methodology, Resources, Visualization, Writing – review & editing. PD-S: Conceptualization, Data curation, Formal Analysis, Project administration, Visualization, Writing – original draft, Writing – review & editing. KH: Conceptualization, Funding acquisition, Project administration, Resources, Supervision, Writing – review & editing.

## References

[1] Merle NS Church SE Fremeaux-Bacchi V Roumenina LT. Complement system part I - molecular mechanisms of activation and regulation. Front Immunol. (2015) 6:262. doi: 10.3389/fimmu.2015.00262 26082779 PMC4451739

[2] Zipfel PF Skerka C. Complement regulators and inhibitory proteins. Nat Rev Immunol. (2009) 9:729–40. doi: 10.1038/nri2620 19730437

[3] Gomez-Arboledas A Acharya MM Tenner AJ. The role of complement in synaptic pruning and neurodegeneration. Immunotargets Ther. (2021) 10:373–86. doi: 10.2147/ITT.S305420 PMC8478425 34595138

[4] Kemper C Köhl J. Novel roles for complement receptors in T cell regulation and beyond. Mol Immunol. (2013) 56:181–90. doi: 10.1016/j.molimm.2013.05.223 23796748

[5] Carroll MC. The role of complement in B cell activation and tolerance. Adv Immunol. (2000) 74:61–88. doi: 10.1016/s0065-2776(08)60908-6 10605604

[6] Kemper C Ferreira VP Paz JT Holers VM Lionakis MS Alexander JJ. Complement: the road less traveled. J Immunol. (2023) 210:119–25. doi: 10.4049/jimmunol.2200540 PMC10038130 36596217

[7] Waters AM Licht C. aHUS caused by complement dysregulation: new therapies on the horizon. Pediatr Nephrol. (2011) 26:41–57. doi: 10.1007/s00467-010-1556-4 20556434 PMC2991208

[8] Smith RJH Appel GB Blom AM Cook HT D’Agati VD Fakhouri F. C3 glomerulopathy - understanding a rare complement-driven renal disease. Nat Rev Nephrol. (2019) 15:129–43. doi: 10.1038/s41581-018-0107-2 PMC6876298 30692664

[9] Schmidt T Afonso S Perie L Heidenreich K Wulf S Krebs CF. An interdisciplinary diagnostic approach to guide therapy in C3 glomerulopathy. Front Immunol. (2022) 13:826513. doi: 10.3389/fimmu.2022.826513 35693785 PMC9186056

[10] Zipfel PF Wiech T Gröne HJ Skerka C. Complement catalyzing glomerular diseases. Cell Tissue Res. (2021) 385:355–70. doi: 10.1007/s00441-021-03485-w PMC8523427 34613485

[11] Wong EKS Kavanagh D. Diseases of complement dysregulation-an overview. Semin Immunopathol. (2018) 40:49–64. doi: 10.1007/s00281-017-0663-8 29327071 PMC5794843

[12] Galindo-Izquierdo M Pablos Alvarez JL. Complement as a therapeutic target in systemic autoimmune diseases. Cells. (2021) 10:148–66. doi: 10.3390/cells10010148 PMC7828564 33451011

[13] Cervia-Hasler C Brüningk SC Hoch T Fan B Muzio G Thompson RC. Persistent complement dysregulation with signs of thromboinflammation in active Long Covid. Science. (2024) 383. doi: 10.1126/science.adg7942 38236961

[14] Dmytrijuk A Robie-Suh K Cohen MH Rieves D Weiss K Pazdur R. FDA report: eculizumab (Soliris) for the treatment of patients with paroxysmal nocturnal hemoglobinuria. Oncologist. (2008) 13:993–1000. doi: 10.1634/theoncologist.2008-0086 18784156

[15] Socié G Caby-Tosi M-P Marantz JL Cole A Bedrosian CL Gasteyger C. Eculizumab in paroxysmal nocturnal haemoglobinuria and atypical haemolytic uraemic syndrome: 10-year pharmacovigilance analysis. Br J Haematol. (2019) 185:297–310. doi: 10.1111/bjh.15790 30768680 PMC6594003

[16] Röth A Rottinghaus ST Hill A Bachman ES Kim JS Schrezenmeier H. Ravulizumab (ALXN1210) in patients with paroxysmal nocturnal hemoglobinuria: results of 2 phase 1b/2 studies. Blood Adv. (2018) 2:2176–85. doi: 10.1182/bloodadvances.2018020644 PMC6134221 30171081

[17] Mastellos DC Hajishengallis G Lambris JD. A guide to complement biology, pathology and therapeutic opportunity. Nat Rev Immunol. (2023) 24:118–41. doi: 10.1038/s41577-023-00926-1 37670180

[18] Hoy SM. Pegcetacoplan: first approval. Drugs. (2021) 81:1423–30. doi: 10.1007/s40265-021-01560-8 34342834

[19] Khan AM Shahnoor S Khan H Affendi M Ghaddar SA. Celebrating a breakthrough: the first-ever FDA-approved treatment for geographic atrophy: a correspondence. Int J Surg. (2023) 109:2559–60. doi: 10.1097/JS9.0000000000000477 PMC10442090 37195786

[20] Liao DS Grossi FV El Mehdi D Gerber MR Brown DM Heier JS. Complement C3 inhibitor pegcetacoplan for geographic atrophy secondary to age-related macular degeneration: A randomized phase 2 trial. Ophthalmology. (2020) 127:186–95. doi: 10.1016/j.ophtha.2019.07.011 31474439

[21] Röth A Barcellini W D’Sa S Miyakawa Y Broome CM Michel M. Sustained inhibition of complement C1s with sutimlimab over 2 years in patients with cold agglutinin disease. Am J Hematol. (2023) 98:1246–53. doi: 10.1002/ajh.26965 37246953

[22] Zipfel PF Wiech T Rudnick R Afonso S Person F Skerka C. Complement inhibitors in clinical trials for glomerular diseases. Front Immunol. (2019) 10:2166. doi: 10.3389/fimmu.2019.02166 31611870 PMC6776600

[23] Jayne DRW Merkel PA Schall TJ Bekker P ADVOCATE Study Group. Avacopan for the treatment of ANCA-associated vasculitis. N Engl J Med. (2021) 384:599–609. doi: 10.1056/NEJMoa2023386 33596356

[24] Novartis receives FDA approval for Fabhalta® (iptacopan), offering superior hemoglobin improvement in the absence of transfusions as the first oral monotherapy for adults with PNH (2023) (Accessed January 12, 2024).

[25] Benamu E Montoya JG. Infections associated with the use of eculizumab: recommendations for prevention and prophylaxis. Curr Opin Infect Dis. (2016) 29:319–29. doi: 10.1097/QCO.0000000000000279 27257797

[26] Girmenia C Barcellini W Bianchi P Di Bona E Iori AP Notaro R. Management of infection in PNH patients treated with eculizumab or other complement inhibitors: Unmet clinical needs. Blood Rev. (2023) 58:101013. doi: 10.1016/j.blre.2022.101013 36117056

[27] Notaro R Luzzatto L. Breakthrough hemolysis in PNH with proximal or terminal complement inhibition. N Engl J Med. (2022) 387:160–6. doi: 10.1056/NEJMra2201664 35830642

[28] Ferreira VP Pangburn MK Cortés C. Complement control protein factor H: the good, the bad, and the inadequate. Mol Immunol. (2010) 47:2187–97. doi: 10.1016/j.molimm.2010.05.007 PMC2921957 20580090

[29] Ripoche J Day AJ Harris TJR Sim RB. The complete amino acid sequence of human complement factor H. Biochem J. (1988) 249:593–602. doi: 10.1042/bj2490593 2963625 PMC1148743

[30] Schmidt CQ Slingsby FC Richards A Barlow PN. Production of biologically active complement factor H in therapeutically useful quantities. Protein Expr Purif. (2011) 76:254–63. doi: 10.1016/j.pep.2010.12.002 PMC4067574 21146613

[31] Weiler JM Daha MR Austen KF Fearon DT. Control of the amplification convertase of complement by the plasma protein beta1H. Proc Natl Acad Sci. (1976) 73:3268–72. doi: 10.1073/pnas.73.9.3268 PMC431003 1067618

[32] Pangburn MK Schreiber RD Müller-Eberhard HJ. Human complement C3b inactivator: isolation, characterization, and demonstration of an absolute requirement for the serum protein beta1H for cleavage of C3b and C4b in solution. J Exp Med. (1977) 146:257–70. doi: 10.1084/jem.146.1.257 PMC2180748 301546

[33] Zipfel PF Hellwage J Friese MA Hegasy G Jokiranta ST Meri S. Factor H and disease: a complement regulator affects vital body functions. Mol Immunol. (1999) 36:241–8. doi: 10.1016/s0161-5890(99)00038-3 10403477

[34] Martín Merinero H Zhang Y Arjona E Del Angel G Goodfellow R Gomez-Rubio E. Functional characterization of 105 factor H variants associated with aHUS: lessons for variant classification. Blood. (2021) 138:2185–201. doi: 10.1182/blood.2021012037 PMC8641096 34189567

[35] Sánchez-Corral P González-Rubio C Rodríguez De Córdoba S López-Trascasa M. Functional analysis in serum from atypical Hemolytic Uremic Syndrome patients reveals impaired protection of host cells associated with mutations in factor H. Mol Immunol. (2004) 41:81–4. doi: 10.1016/j.molimm.2004.01.003 15140578

[36] Zhang Y Ghiringhelli Borsa N Shao D Dopler A Jones MB Meyer NC. Factor H autoantibodies and complement-mediated diseases. Front Immunol. (2020) 11:607211. doi: 10.3389/fimmu.2020.607211 33384694 PMC7770156

[37] Józsi M Strobel S Dahse HM Liu WS Hoyer PF Oppermann M. Anti factor H autoantibodies block C-terminal recognition function of factor H in hemolytic uremic syndrome. Blood. (2007) 110(5):1516–8. doi: 10.1182/blood-2007-02-071472 17495132

[38] Parente R Clark SJ Inforzato A Day AJ. Complement factor H in host defense and immune evasion. Cell Mol Life Sci. (2017) 74:1605–24. doi: 10.1007/s00018-016-2418-4 PMC5378756 27942748

[39] De Córdoba SR De Jorge EG. Translational Mini-Review Series on Complement Factor H: Genetics and disease associations of human complement factor H. Clin Exp Immunol. (2007) 151:1–13. doi: 10.1111/j.1365-2249.2007.03552.x PMC2276932 18081690

[40] Haines JL Hauser MA Schmidt S Scott WK Olson LM Gallins P. Complement factor H variant increases the risk of age-related macular degeneration. Science. (2005) 308:419–21. doi: 10.1126/science.1110359 15761120

[41] Hageman GS Anderson DH Johnson LV Hancox LS Taiber AJ Hardisty LI. A common haplotype in the complement regulatory gene factor H (HF1/CFH) predisposes individuals to age-related macular degeneration. Proc Natl Acad Sci U.S.A. (2005) 102:7227–32. doi: 10.1073/pnas.0501536102 PMC1088171 15870199

[42] Klein RJ Zeiss C Chew EY Tsai J-Y Sackler RS Haynes C. Complement factor H polymorphism in age-related macular degeneration. Science. (2005) 308:385–9. doi: 10.1126/science.1109557 PMC1512523 15761122

[43] Edwards AO Ritter R Abel KJ Manning A Panhuysen C Farrer LA. Complement factor H polymorphism and age-related macular degeneration. Science. (2005) 308:421–4. doi: 10.1126/science.1110189 15761121

[44] Sharma AK Pangburn MK. Biologically active recombinant human complement factor H: synthesis and secretion by the baculovirus system. Gene. (1994) 143:301–2. doi: 10.1016/0378-1119(94)90116-3 8206393

[45] Kühn S Skerka C Zipfel PF. Mapping of the complement regulatory domains in the human factor H-like protein 1 and in factor H1. J Immunol. (1995) 155:5663–70. 7499851

[46] Kerr H Herbert AP Makou E Abramczyk D Malik TH Lomax-Browne H. Murine factor H co-produced in yeast with protein disulfide isomerase ameliorated C3 dysregulation in factor H-deficient mice. Front Immunol. (2021) 12:681098. doi: 10.3389/fimmu.2021.681098 34054871 PMC8149785

[47] Biggs RM Makou E Lauder S Herbert AP Barlow PN Katti SK. A novel full-length recombinant human complement factor H (CFH; GEM103) for the treatment of age-related macular degeneration shows similar *in vitro* functional activity to native CFH. Curr Eye Res. (2022) 47:1087–93. doi: 10.1080/02713683.2022.2053725 35282732

[48] Biggs RM Makou E Lauder S Herbert AP Barlow PN Katti SK. An evaluation of the complement-regulating activities of human complement factor H (FH) variants associated with age-related macular degeneration. Invest Opthalmology Visual Sci. (2022) 63:30. doi: 10.1167/iovs.63.12.30 PMC9716232 36445700

[49] Khanani AM Maturi RK Bagheri N Bakall B Boyer DS Couvillion SS. A phase I, single ascending dose study of GEM103 (Recombinant human complement factor H) in patients with geographic atrophy. Ophthalmol Sci. (2022) 2:100154. doi: 10.1016/j.xops.2022.100154 36249705 PMC9559901

[50] de Guimaraes T Michaelides M. Dry AMD: A review of ongoing, completed and future treatments. Ophthalmol Times Europe. (2023) 19.

[51] Michelfelder S Parsons J Bohlender LL Hoernstein SNW Niederkrüger H Busch A. Moss-produced, glycosylation-optimized human Factor H for therapeutic application in complement disorders. J Am Soc Nephrol. (2017) 28:1462–74. doi: 10.1681/ASN.2015070745 PMC5407710 27932477

[52] Kontermann RE. Strategies for extended serum half-life of protein therapeutics. Curr Opin Biotechnol. (2011) 22:868–76. doi: 10.1016/j.copbio.2011.06.012 21862310

[53] Solá RJ Griebenow K. Glycosylation of therapeutic proteins: an effective strategy to optimize efficacy. BioDrugs. (2010) 24:9–21. doi: 10.2165/11530550-000000000-00000 20055529 PMC2805475

[54] Chia S Tay SJ Song Z Yang Y Walsh I Pang KT. Enhancing pharmacokinetic and pharmacodynamic properties of recombinant therapeutic proteins by manipulation of sialic acid content. Biomedicine Pharmacotherapy. (2023) 163:114757. doi: 10.1016/j.biopha.2023.114757 37087980

[55] Zhou Q Qiu H. The mechanistic impact of N-glycosylation on stability, pharmacokinetics, and immunogenicity of therapeutic proteins. J Pharm Sci. (2019) 108:1366–77. doi: 10.1016/j.xphs.2018.11.029 30471292

[56] Fukuda M Sasaki H Lopez L Fukuda M. Survival of recombinant erythropoietin in the circulation: the role of carbohydrates. Blood. (1989) 73:84–9. doi: 10.1182/blood.V73.1.84.84 2910371

[57] Su D Zhao H Xia H. Glycosylation-modified erythropoietin with improved half-life and biological activity. Int J Hematol. (2010) 91:238–44. doi: 10.1007/s12185-010-0496-x 20131103

[58] Fenaille F Le Mignon M Groseil C Ramon C Riandé S Siret L. Site-specific N-glycan characterization of human complement factor H. Glycobiology. (2007) 17:932–44. doi: 10.1093/glycob/cwm060 17591618

[59] Noris M Galbusera M Gastoldi S Macor P Banterla F Bresin E. Dynamics of complement activation in aHUS and how to monitor eculizumab therapy. Blood. (2014) 124:1715–26. doi: 10.1182/blood-2014-02-558296 PMC4162105 25037630

[60] Aiello S Gastoldi S Galbusera M Ruggenenti P Portalupi V Rota S. C5a and C5aR1 are key drivers of microvascular platelet aggregation in clinical entities spanning from aHUS to COVID-19. Blood Adv. (2022) 6:866–81. doi: 10.1182/bloodadvances.2021005246 PMC8945302 34852172

[61] Yuan X Gavriilaki E Thanassi JA Yang G Baines AC Podos SD. Small-molecule factor D inhibitors selectively block the alternative pathway of complement in paroxysmal nocturnal hemoglobinuria and atypical hemolytic uremic syndrome. Haematologica. (2017) 102:466–75. doi: 10.3324/haematol.2016.153312 PMC5394948 27810992

[62] Schubart A Anderson K Mainolfi N Sellner H Ehara T Adams CM. Small-molecule factor B inhibitor for the treatment of complement-mediated diseases. Proc Natl Acad Sci U.S.A. (2019) 116:7926–31. doi: 10.1073/pnas.1820892116 PMC6475383 30926668

[63] Tortajada A Montes T Martinez-Barricarte R Morgan BP Harris CL de Cordoba SR. The disease-protective complement factor H allotypic variant Ile62 shows increased binding affinity for C3b and enhanced cofactor activity. Hum Mol Genet. (2009) 18:3452–61. doi: 10.1093/hmg/ddp289 PMC3272369 19549636

[64] Lauer N Mihlan M Hartmann A Schlötzer-Schrehardt U Keilhauer C Scholl HPN. Complement regulation at necrotic cell lesions is impaired by the age-related macular degeneration-associated factor-H His402 risk variant. J Immunol. (2011) 187:4374–83. doi: 10.4049/jimmunol.1002488 21930971

[65] Mi Y Lin A Fiete D Steirer L Baenziger JU. Modulation of mannose and asialoglycoprotein receptor expression determines glycoprotein hormone half-life at critical points in the reproductive cycle. J Biol Chem. (2014) 289:12157–67. doi: 10.1074/jbc.M113.544973 PMC4002119 24619407

[66] Koskinen AR Cheng ZZ Pickering MC Kairemo K Meri T Cook HT. Distribution of exogenous complement factor H in mice *in vivo* . Scand J Immunol. (2018) 88. doi: 10.1111/sji.12671 29706017

[67] Shen L Frazer-Abel A Reynolds PR Giclas PC Chappell A Pangburn MK. Mechanistic understanding for the greater sensitivity of monkeys to antisense oligonucleotide-mediated complement activation compared with humans. J Pharmacol Exp Ther. (2014) 351:709–17. doi: 10.1124/jpet.114.219378 25301170

[68] Fakhouri F De Jorge EG Brune F Azam P Cook HT Pickering MC. Treatment with human complement factor H rapidly reverses renal complement deposition in factor H-deficient mice. Kidney Int. (2010) 78:279–86. doi: 10.1038/ki.2010.132 PMC2906702 20445496

[69] Høgåsen K Jansen JH Mollnes TE Hovdenes J Harboe M. Hereditary porcine membranoproliferative glomerulonephritis type II is caused by factor H deficiency. J Clin Invest. (1995) 95:1054–61. doi: 10.1172/JCI117751 PMC441440 7883953

[70] Mastellos DC Yancopoulou D Kokkinos P Huber-Lang M Hajishengallis G Biglarnia AR. Compstatin: a C3-targeted complement inhibitor reaching its prime for bedside intervention. Eur J Clin Invest. (2015) 45:423–40. doi: 10.1111/eci.12419 PMC4380746 25678219

[71] Sahu A Morikis D Lambris JD. Compstatin, a peptide inhibitor of complement, exhibits species-specific binding to complement component C3. Mol Immunol. (2003) 39:557–66. doi: 10.1016/s0161-5890(02)00212-2 12431389

[72] Donadelli R Pulieri P Piras R Iatropoulos P Valoti E Benigni A. Unraveling the molecular mechanisms underlying complement dysregulation by nephritic factors in C3G and IC-MPGN. Front Immunol. (2018) 9:2329. doi: 10.3389/fimmu.2018.02329 30487789 PMC6248175

[73] Trivioli G Vaglio A. The rise of complement in ANCA-associated vasculitis: from marginal player to target of modern therapy. Clin Exp Immunol. (2020) 202:403–6. doi: 10.1111/cei.13515 PMC7670158 32946609

[74] Thurman JM Harrison RA. The susceptibility of the kidney to alternative pathway activation-A hypothesis. Immunol Rev. (2023) 313:327–38. doi: 10.1111/imr.13168 36369971

[75] Noris M Caprioli J Bresin E Mossali C Pianetti G Gamba S. Relative role of genetic complement abnormalities in sporadic and familial aHUS and their impact on clinical phenotype. Clin J Am Soc Nephrol. (2010) 5:1844–59. doi: 10.2215/CJN.02210310 PMC2974386 20595690

[76] Afshar-Kharghan V. Atypical hemolytic uremic syndrome. Hematol Am Soc Hematol Educ Program. (2016) 2016:217–25. doi: 10.1182/asheducation-2016.1.217 PMC6142509 27913483

[77] Zipfel PF Hallström T Hammerschmidt S Skerka C. The complement fitness factor H: role in human diseases and for immune escape of pathogens, like pneumococci. Vaccine. (2008) 26 Suppl 8:I67–74. doi: 10.1016/j.vaccine.2008.11.015 19388168

[78] de Boer ECW van Mourik AG Jongerius I. Therapeutic lessons to be learned from the role of complement regulators as double-edged sword in health and disease. Front Immunol. (2020) 11:578069. doi: 10.3389/fimmu.2020.578069 33362763 PMC7758290

[79] Geerlings MJ de Jong EK den Hollander AI. The complement system in age-related macular degeneration: A review of rare genetic variants and implications for personalized treatment. Mol Immunol. (2017) 84:65–76. doi: 10.1016/j.molimm.2016.11.016 27939104 PMC5380947

[80] Zipfel PF Lauer N Skerka C. The role of complement in AMD. Adv Exp Med Biol. (2010) 703:9–24. doi: 10.1007/978-1-4419-5635-4_2 20711704

[81] Holers VM Banda NK. Complement in the initiation and evolution of rheumatoid arthritis. Front Immunol. (2018) 9:1057. doi: 10.3389/fimmu.2018.01057 29892280 PMC5985368

[82] Magdalon J Mansur F Teles E Silva AL de Goes VA Reiner O Sertié AL. Complement system in brain architecture and neurodevelopmental disorders. Front Neurosci. (2020) 14:23. doi: 10.3389/fnins.2020.00023 32116493 PMC7015047

[83] Mukherjee P Pasinetti GM. Complement anaphylatoxin C5a neuroprotects through mitogen-activated protein kinase-dependent inhibition of caspase 3. J Neurochem. (2001) 77:43–9. doi: 10.1046/j.1471-4159.2001.00167.x 11279260

[84] Pozo-Rodrigálvarez A Li Y Stokowska A Wu J Dehm V Sourkova H. C3a receptor signaling inhibits neurodegeneration induced by neonatal hypoxic-ischemic brain injury. Front Immunol. (2021) 12:768198. doi: 10.3389/fimmu.2021.768198 34975856 PMC8718687

[85] Lee JD Coulthard LG Woodruff TM. Complement dysregulation in the central nervous system during development and disease. Semin Immunol. (2019) 45:101340. doi: 10.1016/j.smim.2019.101340 31708347

[86] Merle DA Provenzano F Jarboui MA Kilger E Clark SJ Deleidi M. mTOR inhibition via rapamycin treatment partially reverts the deficit in energy metabolism caused by FH loss in RPE cells. Antioxidants. (2021) 10:1944. doi: 10.3390/antiox10121944 34943047 PMC8750186

[87] Meri S Haapasalo K. Function and dysfunction of complement factor H during formation of lipid-rich deposits. Front Immunol. (2020) 11:611830. doi: 10.3389/fimmu.2020.611830 33363547 PMC7753009

[88] Chernyaeva L Ratti G Teirilä L Fudo S Rankka U Pelkonen A. Reduced binding of apoE4 to complement factor H promotes amyloid-β oligomerization and neuroinflammation. EMBO Rep. (2023) 24. doi: 10.15252/embr.202256467 PMC10328077 37155564

[89] Yin C Ackermann S Ma Z Mohanta SK Zhang C Li Y. ApoE attenuates unresolvable inflammation by complex formation with activated C1q. Nat Med. (2019) 25:496–506. doi: 10.1038/s41591-018-0336-8 30692699 PMC6420126

[90] Weismann D Hartvigsen K Lauer N Bennett KL Scholl HPN Issa PC. Complement factor H binds malondialdehyde epitopes and protects from oxidative stress. Nature. (2011) 478:76–81. doi: 10.1038/nature10449 21979047 PMC4826616

[91] Kiss MG Papac-Miličević N Porsch F Tsiantoulas D Hendrikx T Takaoka M. Cell-autonomous regulation of complement C3 by factor H limits macrophage efferocytosis and exacerbates atherosclerosis. Immunity. (2023) 56:1809–1824.e10. doi: 10.1016/j.immuni.2023.06.026 37499656 PMC10529786

[92] Andersson J Larsson R Richter R Ekdahl KN Nilsson B. Binding of a model regulator of complement activation (RCA) to a biomaterial surface: surface-bound factor H inhibits complement activation. Biomaterials. (2001) 22:2435–43. doi: 10.1016/S0142-9612(00)00431-2 11511041

[93] Ekdahl KN Fromell K Mannes M Grinnemo K Huber-Lang M Teramura Y. Therapeutic regulation of complement activation in extracorporeal circuits and intravascular treatments with special reference to the alternative pathway amplification loop. Immunol Rev. (2023) 313:91–103. doi: 10.1111/imr.13148 36258635 PMC10092679

[94] Bechtler C Koutsogiannaki S Umnyakova E Hamid A Gautam A Sarigiannis Y. Complement-regulatory biomaterial coatings: Activity and selectivity profile of the factor H-binding peptide 5C6. Acta Biomater. (2023) 155:123–38. doi: 10.1016/j.actbio.2022.10.055 36328123

[95] Wang Z Hood ED Nong J Ding J Marcos-Contreras OA Glassman PM. Combating complement’s deleterious effects on nanomedicine by conjugating complement regulatory proteins to nanoparticles. Advanced Materials. (2022) 34. doi: 10.1002/adma.202107070 PMC9062787 34910334

[96] Connelly C Gibson B Mahendran B Tingle SJ Bates L Cooper M. O043 Evaluation of minimal factor H therapy administered to kidneys during *ex vivo* normothermic perfusion as a treatment to improve ischaemia reperfusion injury. Br J Surg. (2023) 110. doi: 10.1093/bjs/znad101.043

