# Supplementary material for: Moss-produced human complement factor H with modified glycans has an extended half-life and improved biological activity
Source: Front Immunol. 2024 May 10;15:1383123. doi: 10.3389/fimmu.2024.1383123 (PMC11117068; doi:10.3389/fimmu.2024.1383123)
Supplement: Supplementary file 1 [file DataSheet_1.docx]

Supplementary Material

# Materials and methods

*Description of patients*

aHUS patients

Blood samples were collected from aHUS patients/relatives and incubated for 30 min before centrifugation. Serum samples were immediately frozen and stored at –80 °C.

aHUS1 is now a 34-year-old healthy man from an aHUS family carrying a known *CFH* mutation for aHUS (R1215Q, SCR20). In three of his siblings and one of his sons, aHUS occurred before the age of 2 years. Two older siblings died before the approval of eculizumab due to complications of aHUS, dialysis or relapse after transplantation, and one younger sister and his son are under treatment with eculizumab/ravulizumab. The *CFH* mutation causes strong hemolytic activity when serum is incubated with sheep erythrocytes.

aHUS2 is now a 12-year-old boy with a diagnosis of aHUS at the age of 9 due to FH autoantibodies associated with a homozygous CFHR1/3 deletion (DEAP HUS). He received ravulizumab for 1.5 years and mycophenolate mofetil. Ravulizumab was stopped when FH autoantibodies dropped below threshold. At the time of blood collection, the patient was in the acute phase.

aHUS3 is a 48-year-old woman with aHUS preceded by hypertensive crisis and vomiting. Genetic screening revealed no mutation in known complement genes. At the time of blood collection, the patient was in the acute phase.

aHUS4 is a 12-year-old girl with a recent aHUS episode triggered by SARS-CoV-2 infection. Genetic screening revealed a heterozygous pathogenic nonsense variant in CD46 (p.R59X). At the time of blood collection, the patient was in the acute phase.

aHUS5 is a 5-year-old girl with aHUS. Genetic screening revealed a heterozygous intronic VUS (variant of unknown significance) in the *CFH* gene (c.1159+6T>C) predicted *in silico* to modify splicing. At the time of blood collection, the patient was in the acute phase. She underwent full remission following treatment with eculizumab.

aHUS6 is a 41-year-old woman with recent aHUS onset. Genetic screening revealed no mutation in known complement genes. At the time of blood collection, the patient was in the acute phase.

aHUS7 is a 35-year-old man with recent aHUS onset associated with acute hypertensive crisis. Genetic screening revealed a heterozygous VUS in the *ADAMTS13* gene but ADAMTS13 activity was 26% excluding TTP. At the time of blood collection, the patient was in the acute phase with microangiopathic anemia, thrombocytopenia and acute renal failure.

aHUS8 is 74-year-old man with diagnosis of aHUS at the age of 72 years. Genetic screening was performed in another center. At the time of blood collection, the patient was in the acute phase with microangiopathic anemia, thrombocytopenia, low C3 levels and acute renal failure requiring dialysis.

PNH patients

EDTA blood was collected from four different PNH patients and stored at 4 °C.

PNH1 is a 34-year-old male patient under treatment with crovalimab and a CD59-deficient (type III deficiency) erythrocyte count of 90.7%.

PNH2 is a 24-year-old male patient under treatment with ravulizumab and a CD59-deficient (type III deficiency) erythrocyte count of 74.3%.

PNH3 is a 45-year-old female patient under treatment with ravulizumab and a CD59-deficient (type III deficiency) erythrocyte count of 99.9%.

PNH4 is a 53-year-old female patient under treatment with pegcetacoplan and a CD59‑deficient (type III deficiency) erythrocyte count of 47.9%.

*N-glycan analysis by* *HILIC-HPLC-MS*

To assess the identity and quantity of protein linked *N*-glycans, the glycans were liberated from the protein and labeled with procainamide. Briefly, 50-μg aliquots were taken in duplicates from the provided glycoprotein solutions and reduced with DTT in 100 mM ammonium bicarbonate (pH 8.2) at 56 °C for 45 min. Iodoacetamide was added to a final concentration of 25 mM and S-alkylation proceeded in the dark for 30 min. Samples were then precipitated with acetone at –20 °C for 2 h. After washing the pellet with 80% acetone at –20 °C, the samples were dried *in vacuo* and digested with PNGase F overnight. Liberated *N*-glycans were cleaned by passage through a 25-mg C18 Hypersep centrifuge cartridge (Thermo Fisher Scientific, USA) and labeled with procainamide in the presence of cyanoborohydrate at 65 °C for 3 h in the dark. After derivatization, a HILIC SPE cleanup was performed using 50-mg Discovery glycan cartridges (Supelco, USA). Bound derivatized glycans were eluted with 500 µl 20% acetonitrile in water. The glycans were dried *in vacuo* and dissolved in 20 μl water before separation on a Waters (USA) Acquity UPLC Glycan BEH amide column (2.1 × 150 mm, 1.7 μm) with a Security Guard Ultra precolumn (Phenomenex, USA) on a Nexera X2 HPLC system with an RF-20Axs Fluorescence Detector, equipped with a semimicro flow cell (Shimadzu, Japan). Solvent A consisted of 80 mM formic acid, buffered to pH 4.4 with ammonia. Solvent B was 80% acetonitrile in solvent A. The applied gradient started with an initial hold of 99% solvent B for 8 min, decreasing to 57% B in 60 min and 25% B in 2 min, at a flow rate of 0.4 ml/min. The column oven was set to 45 °C and the flow cell thermostat to 40 °C. Fluorescence was measured with excitation at 308 nm and detection at 359 nm. The injection volume was 2.5 μl. Peak identity was assessed by coupling the same HPLC system to a Bruker amaZon speed ETD ion trap mass spectrometer equipped with the standard ESI source. Spectra were recorded in positive ion mode. The percentage content of individual glycans was calculated as the ratio of the corresponding peak area to the total peak area of all glycans.

*Analysis of FH variants by capillary isoelectric focusing (cIEF)*

All three samples and two buffer blanks were analyzed in a master mix containing 0.35% methyl cellulose, 6 M urea, 4% ampholytes (1% pH 2.5–5, 1% pH 5–8 and 2% pH 3–10), 10 mM IDA and pI markers 4.65 and 7.05 with a focus time of 6.5 min (1 min at 1500 V and 5.5 min at 3000 V) using a Maurice C capillary electrophoresis (ProteinSimple, USA) with UV detection. Data were evaluated by pI calibration of the electropherograms using the two internal pI markers and Compass for iCE software (ProteinSimple).

*Analysis of FH variants by circular dichroism (CD) spectroscopy*

FH samples were diluted with phosphate buffer (20 mM K_2_HPO_4_, pH 8.1) to a concentration of ~0.8 g/L. These solutions were used for near-UV CD measurements and further diluted in phosphate buffer to ~0.1 g/L for far-UV CD measurements. Formulation buffer was diluted in phosphate buffer similarly to the samples (1/15 for near-UV, 1/150 for far-UV) and used as a blank. CD measurements were performed in the near and far UV ranges using a Jasco J 1500 spectropolarimeter. Acquired spectra were evaluated using Jasco Software Spectra Manager CFR, J-1500 series, with the following parameters: cell path length 1 cm (near UV), 0.1 cm (far-UV); scan range 350–250 nm (near UV), 250–180 nm (far UV). Blank correction was performed by subtracting buffer spectra from the sample spectra. The blank-corrected sample spectra were then converted to mean residue ellipticity using the actual number of amino acids (1213). FFT noise reduction was applied for far-UV spectra.

*Determination of the extinction coefficient (CPV-101 and sd-FH) by time-resolved amino acid analysis*

Diluted samples were pipetted into glass vials and dried. To ensure high-sensitivity analysis, the glass vials were pyrolyzed at 500 °C for at least 4 h before use. The protein was hydrolyzed to individual amino acids in 6 M HCl and 0.1% phenol at 110 °C in the gas phase. A time-course amino acid analysis was carried out as described in Ph. Eur. 2.2.56 (9.0). Briefly, the protein was hydrolyzed for 24, 48 or 72 h. The hydrolyzed samples were dried and the amino acids were derivatized with the AccQ•Tag fluorophore (Waters) by adding HCl, borate buffer (Waters), norleucine (NLE) as an internal standard (10 pmol/μl), and the AccQ•Tag reagent. The labeled amino acids were separated by high-performance reversed-phase chromatography with fluorescence detection (excitation 250 nm, emission 395 nm) on a Prominence HPLC instrument (Shimadzu) fitted with a Waters XBridge separator column Peptide BEH C18 (2.1 x 150mm). We used AccQ•Tag as eluent A and 60% acetonitrile as eluent B. The labeled amino acids were separated using a multi-step gradient from 4.4% to 42.5% B in 48 min at 25 °C with a flow rate of 0.3 ml/min. Calibration (quantitation and peak assignment by retention time) was achieved using a defined amino acid mixture obtained from NIST with 5–200 pmol for each amino acid. Quantification required an internal amino acid standard (NLE). The data were evaluated using Empower 3 (Waters) and validated using Microsoft Excel. The validated Excel spreadsheets indicated protein concentrations and amino acid compositions based on a linear calibration of amino acid peak areas. The extinction coefficient according to the Lambert-Beer Law was calculated based on the protein content determined by amino acid analysis. The protein UV absorbance (A_280-320_) was measured using a UV-1800 spectrophotometer (Shimadzu) in triplicate against the corresponding buffer blank. The resulting values were averaged to calculate the molar extinction coefficient. BSA was used as a control for the determination of UV absorbance.

*Measurement of FH-C3b binding by surface plasmon resonance (SPR) spectroscopy*

C1 sensor chips were used in a Biacore T200 instrument. C3b was first diluted to 0.05 mg/ml in HBS-P buffer (0.01 M HEPES pH 7.4, 0.15 NaCl, 0.005% Surfactant P20) and subsequently to 1 µg/ml in 10 mM acetate buffer (pH 5.0). The four channels on the sensor chip were activated with 60 µl *N*‑(3-dimethylaminopropyl)-*N*′-ethylcarbodiimide hydrochloride and 60 µl *N*-hydroxysuccinimide at 10 µl/min for 420 s until all channels registered at least 70 response units (RU). C3b solution was passed through three of the channels at 5 µl/min until the RU increased to ~150, while the other channel was used as a blank reference. After C3b immobilization, ethanolamine was passed through all four channels for 420 s at a flow rate of 10 µl/min. Serial dilutions of sd-FH, CPV101 and CPV104 (250, 125, 62.5, 31.3, 15.6, 7.8, 3.9 and 2 pM) were prepared in HBS-P buffer and passed through the system at 30 µl/min with a contact time of 60 s and a dissociation time of 300 s. The sample compartment and analysis temperature was 10 °C. Kinetic parameters were calculated using Biacore T200 evaluation software.

*Cofactor Activity*

2 µg C3b and 0.5 µg FI were incubated with increasing concentrations (0.5, 1, 2, 10 nM) of sd-FH, CPV-104 or CPV-101 at 37 °C for 30 minutes in a total volume of 20 µl. The proteolytic cleavage of C3b catalyzed by FH and FI was analyzed by SDS-PAGE on 8% gels followed by Coomassie staining. To quantify C3b cleavage products the gel analysis tool from ImageJ was used. Band intensities of C3b-α, the α68 as well as the α46 and α43 fragments were normalized against the C3b-β chain.

*Decay Acceleration Assay*

2,5 µg C3b was coated overnight on NUNC MaxiSorb plates (44-2404; ThermoFisher) overnight at 4 °C. The C3bBb complex was generated by adding 400 ng FB (A135, Complement Technology) and 25 ng factor D (A136, Complement Technology) in phosphate buffer containing 2 mM nickel chloride, 25 mM sodium chloride, and 4% BSA. After incubation for 2 hours at 37 °C and washing, increasing concentrations (1, 5, 10 nM) of CPV-104, CPV-101, sd-FH or BSA were added to the convertase complexes for 30 minutes at 37 °C. FH-mediated dissociation of the convertase was monitored by determination of leftover C3bBb complexes by measuring the amount of C3b-bound Bb detected by a FB-specific antibody (341272, Calbiochem, 1:2.000 in PBS and 0.1% BSA) followed by a HRP-conjugated rabbit anti-goat (P0449, Dako, 1:10.000 in PBS and 0.1% BSA) secondary antibody. After washing, TMB Substrate was added, and the OD at 450 nm determined.

*C3b binding Assay*

1 μg C3b was coated on MaxiSorb plates overnight at 4 °C in PBS. Unspecific binding sites were blocked with 2% BSA (Thermo Scientific) in PBS for 2 hours at 37 °C. Wells were washed with assay buffer (20 mM HEPES, 130 mM NaCl, and 0.1% Tween20; pH 7.3), and unspecific binding sites were blocked with 1% BSA in assay buffer for 2 hours at 37 °C. After washing, increasing concentrations (1, 5, 10 nM) of CPV-104, CPV-101, sd-FH or BSA were added to the wells and incubated for 1 h at 37 °C. After washing, bound FH was detected using a goat anti-human FH antibody (A237, Complement Technology, 1:10.000 in PBS and 0.1% BSA) followed by incubation with an HRP-conjugated rabbit anti-goat serum (P0449, Dako, 1:10.000 in PBS and 0.1% BSA). After washing, TMB Substrate was added, and the OD at 450 nm determined.

*Hemolysis Assay*

Briefly, 5×107 freshly prepared sheep erythrocytes (31100100, Fiebig, Idar-Oberstein, Germany) were diluted to a final volume of 25 μl in GVB-MgEGTA buffer. CPV-104, CPV-101 or sd-FH was diluted to a final volume of 15 μl at various concentrations (0, 5, 10, 50, 100, 500, 1000 nM) in GVB-MgEGTA buffer and added to the erythrocytes. The hemolytic reaction was started by adding 10 μl of aHUS1 serum to the reaction. The mixture was incubated at 37°C for 30 minutes and stopped by adding 200 μl GVB-EDTA buffer. After centrifugation, supernatants were transferred into a 96 well plate and the OD of the supernatants was measured at 414 nm.

*^125^I-radiolabeling to study exposure, tissue distribution and elimination in wild-type mice*

Radiolabeling and *in vivo* studies using ^125^I-labeled material were carried out at Chelatec SAS (France). Animal experiments were performed in strict accordance with European Union Directive 2010/63/EU. All experimental procedures were approved by The French Ministry of Higher Education, Research (Apafis-27745) and the local ethical committee C2EA-06 (Comité d’Ethique en Expérimentation Animale des Pays de la Loire). The three FH variants were labeled with ^125^I on NH_2_ groups using N-succinimidyl 3-^125^I-iodobenzoate (^125^I-SIB). Briefly, a tube containing 1.65 mCi (61 MBq, 0.75 nmol) of ^125^I-SIB was mixed with 400 μg of FH protein and ~130 µL of PBS (pH 7.4) before adjusting the pH to 8.0 and stirring gently for 30 min at room temperature. The unreacted ^125^I-SIB was removed on a PD-10 desalting column and the radiochemical purity (ITLC/10% TCA precipitation) and total activity in the resulting protein solution were determined. The quality of radiolabeled proteins was assessed by radio SE-HPLC and SDS-PAGE. Radiochromatograms of each ^125^I-SIB-labeled FH variant showed the presence of one major peak (>98%) corresponding to the FH monomer. Electrophoretic profiles obtained after staining with Coomassie Brilliant Blue were identical for unlabeled and ^125^I-SIB-labeled FH variants confirming that the radiolabeling process did not cause protein degradation. Dosing solutions were prepared by diluting labeled CPV-101, CPV-104 and sd-FH with each unlabeled protein to obtain a specific activity of ~0.025 mCi/mg (0.925 MBq/mg) and 6.7 mg/mL for the FH concentration.

CD-1 mice (n = 3) about 7 weeks old (25–32 g) were anesthetized and injected intravenously via the retro-orbital plexus at a dose of 40 mg/kg (0.93–1.19 MBq). At the indicated time points, 60–100 µl of blood was sampled and, at the terminal time point, the blood samples were obtained from exsanguination via intracardiac puncture following the intraperitoneal injection of a mixture of ketamine and xylazine. Blood samples were collected into pre-weighed Microvette tubes with heparin lithium (Sarstedt, Germany) and processed for plasma (centrifugation for 5 min at 2000 *g*). Plasma samples were analyzed for radioactivity in a gamma counter. The radioactivity in plasma samples was expressed as a percentage of the injected dose per gram (%ID/g). For each compound, the half-life was calculated from the early time points (2 min to 6 h) considering a one-compartment model system.

At terminal time points (30 min, 2, 6, 24 and 48 h post injection), mice were euthanized as described above and organs of interest were harvested, rinsed in 0.9% NaCl, placed in vials, weighed and radioactivity was measured using the gamma counter. The selected tissues/organs were the bladder (empty), liver, pancreas, spleen, kidneys, lungs, heart, gastro-intestinal tract (stomach, small intestine, and colon with contents), brain, skeletal muscle, thyroid, head and tail. The kidneys were counted separately. The liver was counted in its entirety by cutting it into two pieces and counting the pieces individually. The concentration of radioactivity was expressed as a percentage of the injected dose per gram of organ (%ID/g).

# Supplementary figures and tables

## Supplementary figures


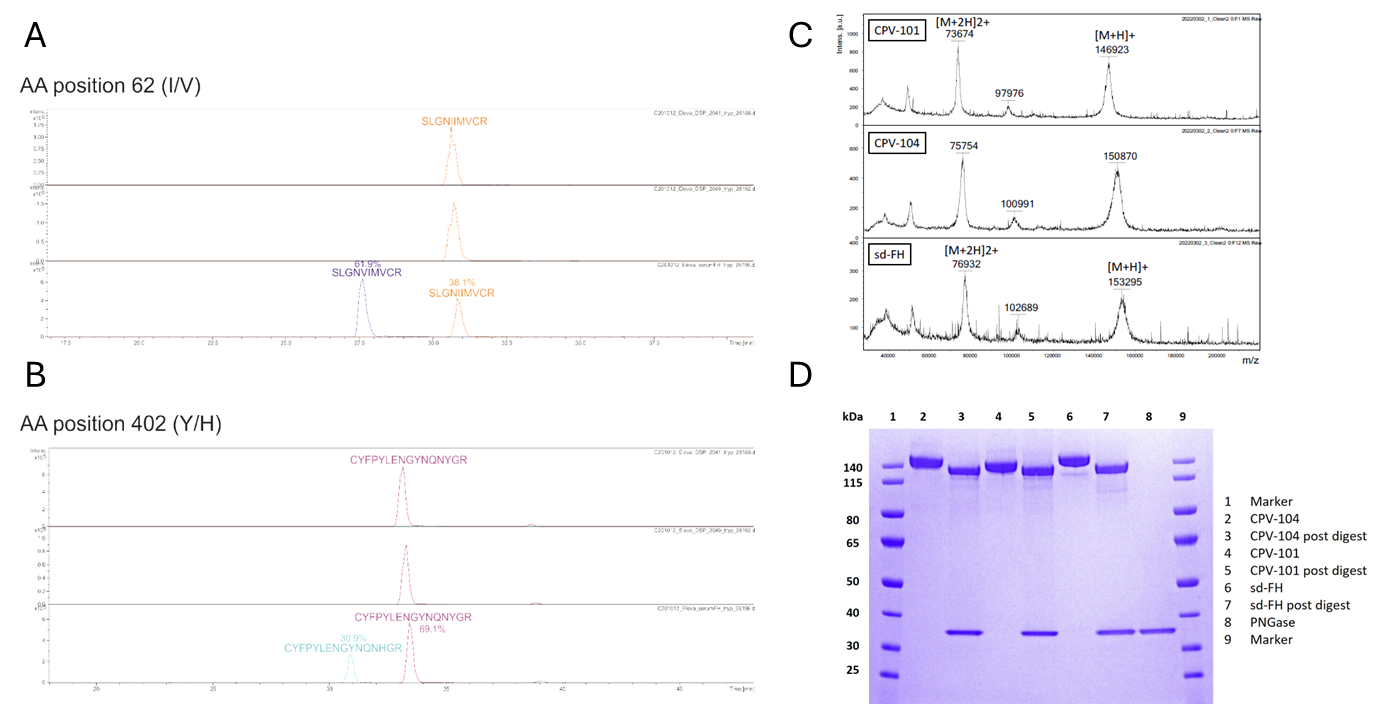


**Supplementary Figure S1.** A) Upper two traces show two independent CPV-101 batches containing only isoleucine at position 62. Lower trace represents commercially available sd-FH with a mixture of valine and isoleucine at position 62. B) Upper two traces show two independent CPV-101 batches containing only tyrosine at position 402. Lower trace represents commercially available sd-FH with a mixture of tyrosine and histidine at position 402. C) Intact mass spectra of CPV-101, CPV-104 and sd-FH. D) SDS-PAGE of CPV-101, CPV-104 and sd-FH before and after digestion with PNGaseF. Post-digest bands of all variants show the same in-gel mobility, indicating that the differences in molecular weight between the variants originate from different glycosylation patterns.

**
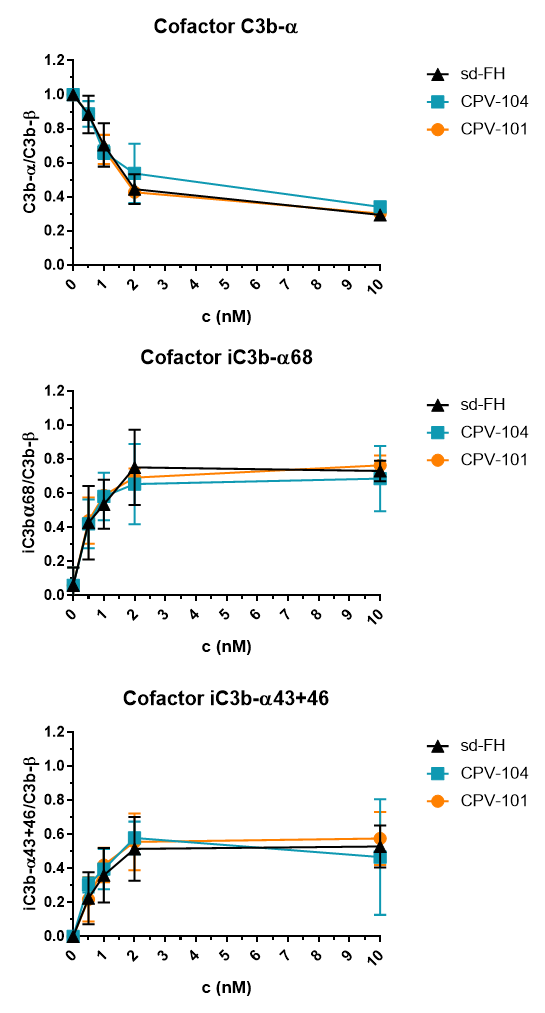
**

**Supplementary Figure S2.** Analysis of C3b cleavage products in cofactor activity assay from Figure 3A in main manuscript: C3b and FI were incubated with increasing concentrations (0.5, 1, 2, 10 nM) CPV104, CPV-101 an sd-FH at 37 °C for 30 min. After SDS-PAGE and Coomassie staining, the C3b cleavage products were quantified using the gel analysis tool from ImageJ. Band intensities of C3b-alpha, the iC3b-alpha68 as well as the alpha-46 and alpha-43 fragments were normalized against the C3b-beta chain. Results are shown as means +- SD from 3 independent experiments.

**Supplementary Figure S3.** Organ/blood ratio 30 min after the intravenous injection of ^125^I-labeled FH variants into wild-type mice at a dose of 40 mg/kg (n=3).


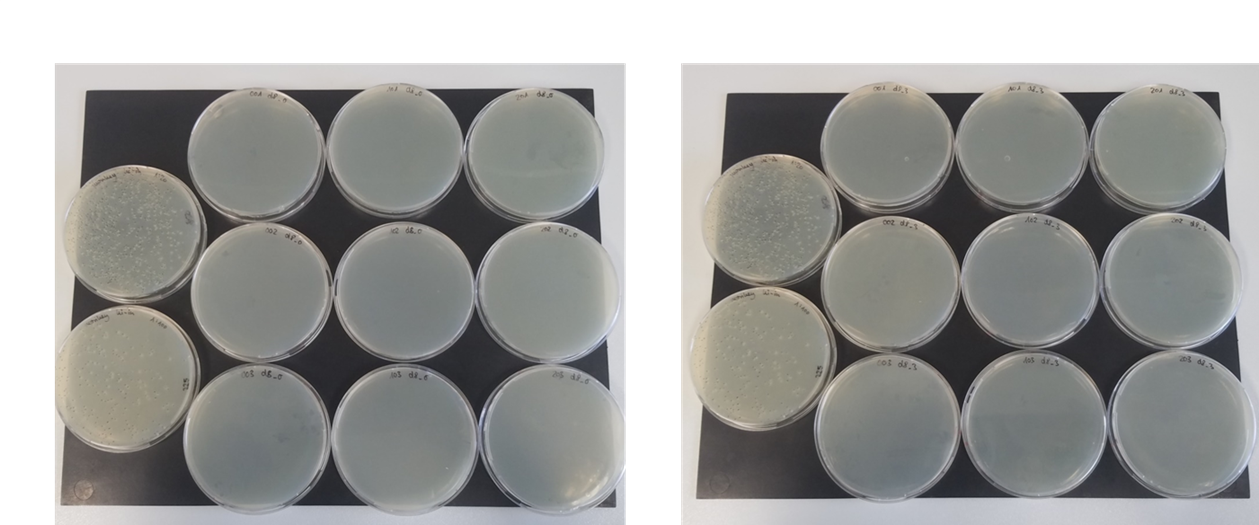


**Supplementary Figure S4**: The ability of CPV-104 to inhibit the growth of *E. coli* in the serum of cynomolgus monkeys was analyzed by collecting serum samples before (left) and 3 h after (right) a single injection of CPV-104. Serum was incubated with *E. coli* cells for 1 h at 37 °C and plated on agar for overnight incubation at 37 °C. No colonies could be detected when *E. coli* cells were incubated with monkey serum before and after CPV-104 treatment, whereas bacterial growth was evident in heat inactivated serum (two plates on the farthest right in both sets).

**Table S1** Yields of single purification steps in a representative purification process, partial harvest of 500L Reactor.

| **Step** | **FH [g/L]** | **Volume [L]** | **FH [g]** | **Total yield [%]** |
| --- | --- | --- | --- | --- |
| Concentrated Harvest | 12.5 | 2.8 | 35 | 100% |
| FH-affinity column, Pool | 2.5 | 13.3 | 33 | 94% |
| In vitro sialylation | 8.4 | 3.2 | 27 | 78% |
| Capto DeVirs column, Pool | 2.1 | 11.4 | 24 | 69% |
| Capto Adhere ImpRes column, Pool | 1.0 | 21.9 | 21 | 60% |
| Final concentration | 22.0 | 0.9 | 19 | 54% |

**Table S2.** Comparison of main glycoforms (%peak area of HILIC-HPLC-FLD chromatograms) between three FH variants.

| **Glycan structure** | **%Peak Area** | | |
| --- | --- | --- | --- |
|  | **CPV-104** | **CPV-101** | **sd-FH** |
| NaM*2Me | 3 | - | - |
| NaM*1Me | 5 | - | - |
| NaM | 10 | - | - |
| NaA | 10 | - | 14 |
| Na(FA) | 12 | - | - |
| NaNa | 53 | - | 56 |
| NaNaNa | - | - | 9 |
| GnM*2Me | - | 4 | - |
| GnM*1Me | - | 7 | - |
| GnM | - | 10 | - |
| GnGn | - | 54 | - |
| Gn(FA) | - | 13 | - |
| others* | 8 | 16 | 21 |

**Table S3. C**omparison of affinity constants (SPR measurements) between sd-FH and two polymorphic variants of CPV-101. CPV-101 Val62/Hys402 showed a similar binding capacity to sd-FH whereas CPV-101 Ile62/Tyr402 showed a higher binding capacity than sd-FH.

| **Sample** | **K_D_ (M)** |
| --- | --- |
| sd-FH | 5.46 ×10^-8^ |
| CPV-101 Val62/Hys402 | 5.48 ×10^-8^ |
| CPV-101 Ile62/Tyr402 | 3.48 ×10^-8^ |
| Ratio sd-FH/CPV-101 Val62/Hys402 | 1.00 |
| Ratio sd-FH/CPV-101 Ile62/Tyr402 | 1.57 |
